# TECPR1 promotes aggrephagy by direct recruitment of LC3C autophagosomes to lysosomes

**DOI:** 10.1038/s41467-020-16689-5

**Published:** 2020-06-12

**Authors:** Lisa Wetzel, Stéphane Blanchard, Sowmya Rama, Viola Beier, Anna Kaufmann, Thomas Wollert

**Affiliations:** 1 Membrane Biochemistry and Transport, UMR3691 CNRS, Institute Pasteur, 28 rue du Dr Roux, 75015 Paris, France; 2 Molecular Membrane and Organelle Biology, Max Planck Institute of Biochemistry, Am Klopferspitz 18, 82152 Martinsried, Germany

**Keywords:** Macroautophagy, Membrane trafficking

## Abstract

The accumulation of protein aggregates is involved in the onset of many neurodegenerative diseases. Aggrephagy is a selective type of autophagy that counteracts neurodegeneration by degrading such aggregates. In this study, we found that LC3C cooperates with lysosomal TECPR1 to promote the degradation of disease-related protein aggregates in neural stem cells. The N-terminal WD-repeat domain of TECPR1 selectively binds LC3C which decorates matured autophagosomes. The interaction of LC3C and TECPR1 promotes the recruitment of autophagosomes to lysosomes for degradation. Augmented expression of TECPR1 in neural stem cells reduces the number of protein aggregates by promoting their autophagic clearance, whereas knockdown of LC3C inhibits aggrephagy. The PH domain of TECPR1 selectively interacts with PtdIns(4)P to target TECPR1 to PtdIns(4)P containing lysosomes. Exchanging the PH against a tandem-FYVE domain targets TECPR1 ectopically to endosomes. This leads to an accumulation of LC3C autophagosomes at endosomes and prevents their delivery to lysosomes.

## Abstract (web summary)

Many neurodegenerative disorders are characterised by the accumulation of protein aggregates in neurons. Here, the authors show that the lysosomal protein TECPR1 selectively recruits mature autophagosomes via an interaction with LC3C to break down protein aggregates in neural stem cells.

## Introduction

Macroautophagy (autophagy in the following) is a highly conserved cellular recycling pathway that sequesters cytoplasmic material and delivers it to lysosomes for degradation^1,2^. A unique Ub-like conjugation system promotes the conjugation of six human autophagy related 8 proteins (hATG8s) to the lipid phosphatidylethanolamine within autophagic membranes^3,4^. These hATG8s are structurally related to Ub^5^. The family members LC3A, LC3B and LC3C are coordinating the expansion of phagophores (also called isolation membranes), whereas GABARAP, GABARAPL1 and -L2 mostly regulate later steps in autophagy^6–8^. Their conjugation requires the sequential action of E1-like ATG7, E2-like ATG3 and E3-like ATG12–ATG5–ATG16L1 enzymes^3,9^. Interestingly, ATG12–ATG5 does not only interact with ATG16L1 to promote phagophore expansion, it also binds TECPR1 (Tectonin beta-propeller repeat-containing protein) through a common ATG5-interacting motif (AIR)^10^.

TECPR1 possesses two WD-repeat domains (TR), two dysferlin domains, an unstructured region and a PH domain^11^. The latter binds phosphatidylinositol-3-phosphate (PtdIns(3)P), which is present in phagophores and autophagosomes^12,13^. TECPR1 localizes to lysosomal membranes and depletion of TECPR1 leads to an accumulation of p62 and LC3. Furthermore, TECPR1 expression is downregulated in neurons of patients suffering from TDP-43 proteinopathies including amyotrophic lateral sclerosis and frontotemporal dementia^14,15^. This suggests that the function of TECPR1 in late steps of autophagy promotes clearance of protein aggregates^12,16^. The molecular function of TECPR1 during late steps of autophagy remains, however, elusive.

Here we demonstrate that deletion of TECPR1 leads to a selective accumulation of LC3C-positive autophagosomes that colocalize with Ub, the selective autophagy receptor p62 and the late autophagic marker STX17. TECPR1 possesses a LIR motif that binds all hATG8s and an N-terminal beta-propeller (TR1) domain that selectively interacts with LC3C. Moreover, TECPR1 binds PtdIns(4)P in vitro and in vivo. Targeting TECPR1 ectopically to PtdIns(3)P-positive membranes impairs trafficking and degradation of LC3C autophagosomes. Strikingly, overexpression of TECPR1 in neural stem cells (NSCs) diminishes the accumulation of neurotoxic protein aggregates such as Huntingtin (Htt) in an autophagy-dependent manner, whereas depletion of LC3C augments aggregation of proteins. Our results thus show that the cooperation of LC3C and TECPR1 promotes clearance of protein aggregates in NSCs by selective autophagy.

## Results

### TECPR1 binds LC3C selectively

**Fig. 1:**
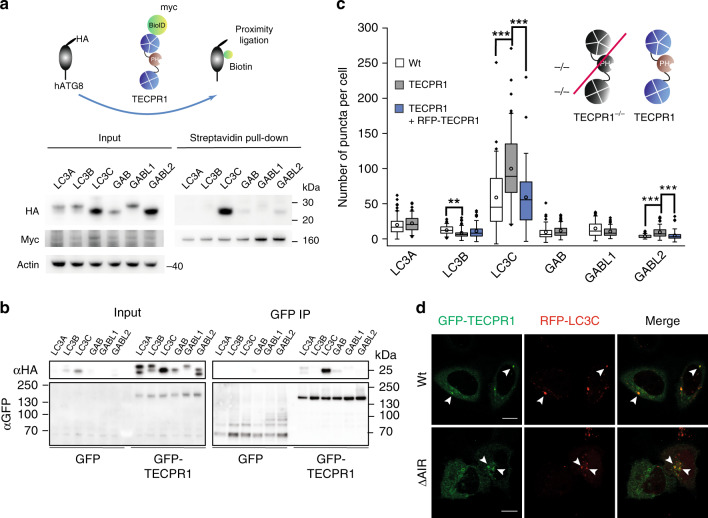
TECPR1 binds LC3C independently of ATG12–ATG5. **a** BioID assay of TECPR1^KO^ cells cotransfected with MycBioID-TECPR1 and HA-tagged hATG8s. Corresponding cell lysates were precipitated using streptavidin beads and samples were immunoblotted with the indicated antibodies. GAB = GABARAP. **b** Immunoprecipitation (IP) of GFP-TECPR1 or GFP and co-IP of HA-LC3C from lysates of cells expressing HA-LC3C and GFP-TECPR1 or GFP. Actin served as loading control. Western blots of lysates and co-IPs as indicated. **c** The number of GFP-hATG8 puncta was counted in starved wild-type (wt) or TECPR1^KO^ cells as well as in TECPR1^KO^ cells transfected with RFP-TECPR1 (*n* = 47 cells pooled from three independent experiments). Box plots represent the first (25%) and third (75%) quartiles, respectively. The center line represents the median, whiskers the standard deviation and minima and maxima are available from the Source Data file. *P*-values were calculated using a two-tailed Student’s *t*-test (**P < 0.01, ***P < 0.001). **d** Confocal images of TECPR1^KO^ cells coexpressing GFP-TECPR1 or GFP-TECPR1^ΔAIR^ and RFP-LC3C. Both TECPR1 and TECPR1^ΔAIR^ colocalized with LC3C. Scale bars, 10 µm. Source data are provided as a Source Data file.

Previous studies suggested that the lysosomal protein TECPR1 coordinates final steps in autophagy by promoting the fusion of autophagosomes with lysosomes^12^. To reveal whether TECPR1 selectively interacts with hATG8 members to promote the fusion of autophagosomes with lysosomes, we applied a proximity-dependent biotin identification (BioID) approach^17^. We therefore co-expressed HA-tagged hATG8s with BioID-TECPR1 and analyzed biotinylation of hATG8s in cell lysates. Interestingly, LC3C was strongly biotinylated, whereas no significant biotinylation of the other hATG8s was observed (Fig. 1a and Supplementary Fig. 1a). We next tested whether TECPR1 interacts with LC3C by performing co-immunoprecipitation (co-IP) experiments using GFP-TECPR1 or GFP as bait. We found that TECPR1 selectively interacts with HA-LC3C, whereas no significant binding of other hATG8s was detected (Fig. 1b). We next systematically compared the number of GFP-hATG8 puncta in wild-type and TECPR1-knockout^(KO)^ cells (Supplementary Table 1). Interestingly, we observed a significant accumulation of LC3C puncta in TECPR1^KO^ cells (Fig. 1c and Supplementary Fig. 1b, c). We concluded from these experiments that the selective interaction of TECPR1 with LC3C promotes the lysosomal degradation of LC3C vesicles.

To prove this hypothesis, we tested whether TECPR1 colocalizes with LC3C at lysosomes. Consistent with a previous study^12^, we found that GFP-TECPR1 puncta colocalized with the late endosomal/lysosomal markers Rab7 and LAMP2 and with the recycling endosomal marker Rab11A, but not with the early endosomal marker EEA1 (Supplementary Fig. 1d, e). Moreover, TECPR1 was identified on autolysosomes in electron micrographs using correlative light electron microscopy (CLEM) (Supplementary Fig. 1e). A second population of TECPR1 was found on smaller vesicles with apparent diameters of ~200 nm (Supplementary Fig. 1f). We next investigated whether TECPR1 and LC3C colocalize in vivo by coexpressing GFP-TECPR1 with RFP-LC3C. Consistent with our BioID experiment, a strong colocalization of both proteins was observed (Fig. 1d). Finally, we performed CLEM of RFP-LC3C and GFP-TECPR1-positive puncta and observed that electron-dense LC3C-positive vesicles are in immediate vicinity of TECPR1-positive autolysosomes (Supplementary Fig. 1g). Collectively, our results thus suggest that TECPR1 selectivity interacts with LC3C to facilitate the lysosomal degradation of LC3C vesicles.

A previous study reported that TECPR1 possesses an ATG5-interacting region (AIR)^12^ that promotes the formation of a lysosomal ATG12–ATG5–TECPR1 complex. To test whether TECPR1 cooperates with ATG12–ATG5 to recruit LC3C, we deleted the AIR motif in TECPR1 and investigated the colocalization of the corresponding TECPR1^∆AIR^ mutant with LC3C in TECPR1^KO^ cells. We found that GFP-TECPR1^∆AIR^ still strongly colocalized with LC3C (Fig. 1d), demonstrating that TECPR1 recruits LC3C independently of ATG12–ATG5.

### TECPR1 possesses an LC3-interacting motif

**Fig. 2:**
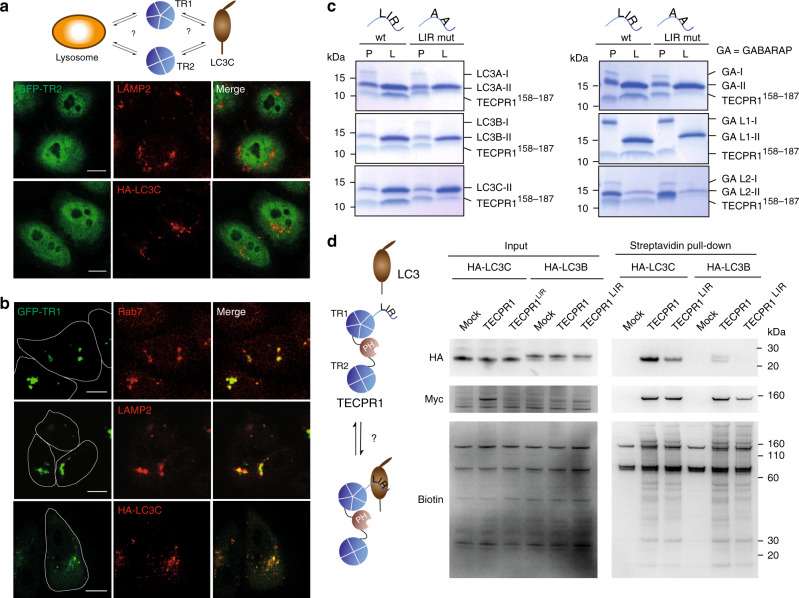
TECPR1 possesses an LC3-interacting motif. **a** Colocalization of immunostained LAMP2 and HA-LC3C with GFP-tagged TR2 in starved TECPR1^KO^ cells. Scale bars, 10 µm. **b** Colocalization of immunostained Rab7, LAMP2 and HA-LC3C with GFP-tagged TR1 in starved TECPR1^KO^ cells. Scale bars, 10 µm. **c** Floatation assay of small unilamellar vesicles to which the indicated hATG8 variant was conjugated with a peptide (TECPR1^158–187^) that contained the W^175^xxI^178^ motif or its corresponding A^175^xxA^178^ mutant (LIR mut). The membrane-free protein fractions (P) and the floating liposome fractions (L) were collected and analyzed by SDS-PAGE. **d** BioID assay of TECPR1^KO^ cells transfected with the empty vector pLPCX (mock), pLPCX-MycBioID-TECPR1 (TECPR1) or pLPCX-MycBioID-TECPR1^W175A/I178A^ (TECPR1^LIR^), and cotransfected with either HA-LC3C or HA-LC3B. Corresponding cell lysates were precipitated using streptavidin beads and samples were immunoblotted with the indicated antibodies. Source data are provided as a Source Data file.

The direct and selective interaction of TECPR1 and LC3C in vivo suggests that TECPR1 possesses a distinct LC3C binding site. To identify this site, we first expressed TR1 and TR2 independently in TECPR1^KO^ cells and investigated their localization and their capacity to recruit LC3C. The C-terminal TR2 domain was mostly distributed in the cytosol of TECPR1^KO^ cells (Fig. 2a). By contrast, TR1 was localizing to Rab7- and LAMP2-positive structures (Fig. 2b). Furthermore, TR1 colocalized with LC3C to a similar degree as TECPR1 (Supplementary Fig. 2a, b). We concluded that the TR1 domain targets TECPR1 to lysosomes and possesses an LC3C selective binding motif.

Many proteins that bind hATG8s contain a conserved peptide motif, termed LC3-interacting region (LIR)^18^. By analyzing the sequence of TR1, we identified the potential LIR W^175^AKI^178^. To test whether this motif selectively interacts with LC3C, we conjugated hATG8s to small unilamellar vesicles (SUVs) and performed cofloatation assays using a peptide that contained the LIR. We recovered this peptide from all fractions that contained hATG8-SUVs, but not from those that lacked hATG8s (Fig. 2c). This demonstrates that although the LIR W^175^AKI^178^ interacts with hATG8s, it is promiscuous and does not selectively recognize LC3C. To confirm the specificity of the interaction, we mutated residues W^175^ and I^178^ to alanine (A^175^AKA^178^) and repeated floatation experiments. The amount of the A^175^AKA^178^ peptide that was recovered from membrane fractions was strongly reduced (Fig. 2c), showing that the interaction of the LIR W^175^AKI^178^ with hATG8s is specific.

In order to characterize the functional importance of the identified LIR in vivo, we applied a BioID assay by coexpressing MycBioID-TECPR1^wt^ or the corresponding LIR mutant (MycBioID-TECPR1^W175A/I178A^) with HA-LC3C in TECPR1^KO^ cells. We found that HA-LC3C was strongly biotinylated in cells expressing MycBioID-TECPR1^wt^ but only weakly if MycBioID-TECPR1^W175A/I178A^ was expressed (Fig. 2d). Moreover, the weak yet detectable biotinylation of LC3B was further decreased in cells expressing the LIR mutant MycBioID-TECPR1^W175A/I178A^. Finally, co-immunoprecipitation of LC3B or LC3C with TECPR1^wt^ or TECPR1^W175A/I178A^ confirmed that the LIR motif is important but not essential for LC3C binding (Supplementary Fig. 2c). Our data thus show that the selectivity of TECPR1 towards LC3C requires a cooperation of its promiscuous LIR with other regions in TECPR1, most likely localized within the TR1 domain.

### TECPR1 recruits LC3C vesicles to lysosomes

**Fig. 3:**
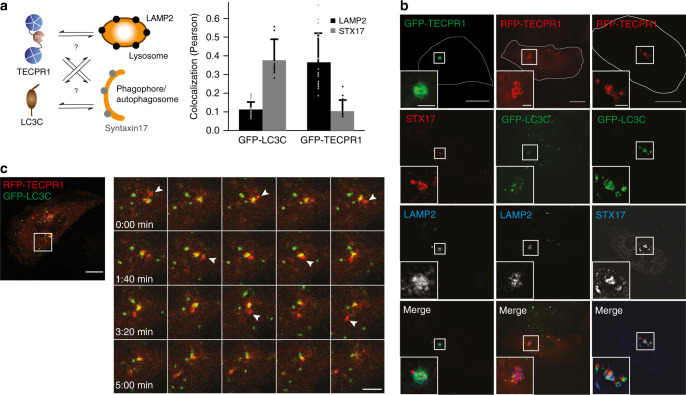
TECPR1 recruits LC3C vesicles to lysosomes. **a** Quantitative analysis of the colocalization of GFP-LC3C and GFP-TECPR1 with immunostained LAMP2 and STX17 in starved HeLa cells (*n* = 20 cells pooled from three independent experiments). Data are represented as mean ± SD. **b** Structured-illumination microscopy of starved HeLa cells expressing GFP-TECPR1 or RFP-TECPR1 together with GFP-LC3C as indicated. Cells were fixed and immunostained for LAMP2 or STX17. LC3C puncta are in the immediate vicinity of TECPR1/LAMP2 compartments. Scale bars, 10 µm, insets, 2.5 µm. **c** Time-lapse video microscopy of TECPR1^KO^ cells coexpressing RFP-TECPR1 and GFP-LC3C. Confocal images were captured every 10 s and every other frame is shown. Arrows indicate moving structures that transiently interact. Scale bar, 10 µm, insets, 2.5 µm. See also Supplementary Movies 1, 2, 3 and 4. Source data are provided as a Source Data file.

The proximal subcellular localization of LC3C and TECPR1 and their direct interaction raise the question whether LC3C and TECPR1 interact in *cis* at the same or in *trans* at two distinct membranes. To address this question, we co-expressed RFP-TECPR1 and GFP-LC3C in HeLa cells and analyzed their colocalization with the lysosomal marker LAMP2 or with the late autophagic marker Syntaxin 17 (STX17). Interestingly, TECPR1 mainly colocalized with LAMP2, whereas a strong colocalization of LC3C with STX17 but not with LAMP2 was observed (Fig. 3a and Supplementary Fig. 3a). Of note, many STX17-positive LC3C puncta were in close proximity to LAMP2-positive TECPR1 structures. Since STX17 is only present at completed and fully sealed autophagosomes^8^, our results suggest that LC3C vesicles correspond to completed autophagosomes.

To further characterize this apparent juxtaposition, we analyzed the colocalization of TECPR1 and LC3C with LAMP2 and STX17 by structured-illumination microscopy. We indeed observed that GFP-TECPR1 and LAMP2 colocalized at big structures, whereas GFP-LC3C was present on small puncta proximal to RFP-TECPR1 (Fig. 3b and Supplementary Movie 1). Moreover, expression of GFP-TECPR1 and immunostaining for endogenous LC3 family proteins revealed a similar pattern. Small LC3-positive puncta were in juxtaposition with GFP-TECPR1 and LAMP2-positive structures (Supplementary Fig. 3b).

To investigate whether membrane bound TECPR1 indeed recruits LC3C vesicles, we reconstituted the system with purified proteins on model membranes. In a first step, we conjugated Alexa488-labeled LC3C to SUVs using the purified human Ub-like conjugation system (Supplementary Fig. 3c). These SUVs represent LC3C vesicles that were observed in the vicinity of TECPR1 structures. In a second step, we decorated GUVs with TECPR1 in order to mimic lysosomal TECPR1-positive compartments. After coincubation of both membrane populations, we indeed observed a pronounced clustering of LC3C-SUVs at TECPR1-positive GUVs. However, in the absence of TECPR1, GUVs failed to recruit LC3C-SUVs (Supplementary Fig. 3d). Our experiments thus demonstrate that TECPR1 recruits LC3C vesicles in vitro.

We next aimed to characterize the dynamic interaction of LC3C and TECPR1 compartments in time and space by following trafficking of LC3C to TECPR1 in living cells. One fraction of LC3C puncta (16 ± 2%) was tethered to TECPR1 structures (>1.0 µm in diameter), whereas the second population was in transient contact (Fig. 3c and Supplementary Movie 2). We next tested, whether TECPR1 compartments correspond to acidified lysosomes using lysotracker deep red. We found that 25% of lysotracker positive structures colocalize with TECPR1. Theses lysosomes appeared to be larger (>1 µm) and less mobile than structures that did not colocalize with TECPR1. Interestingly, we occasionally observed ring-like GFP-TECPR1 structures that contained dot-like lysotracker staining, which is consistent with a mainly (auto)lysosomal localization of TECPR1 (Supplementary Fig. 3e and Movie 3). We next followed trafficking of LC3C vesicles to acidic compartments in lysotracker-treated cells that expressed GFP-LC3C. We again observed two populations of LC3C puncta. One fraction of puncta with diameters >0.5 µm colocalized with immobile and large (<1 µm) lysotracker structures. However, the majority of LC3C puncta (85%) was smaller and interacted with acidified compartments transiently (Supplementary Fig. 3f and Movie 4). Our data thus show that LC3C puncta are either tethered to or in transient contact with TECPR1 compartments.

### TECPR1 selectively binds PtdIns(4)P

**Fig. 4:**
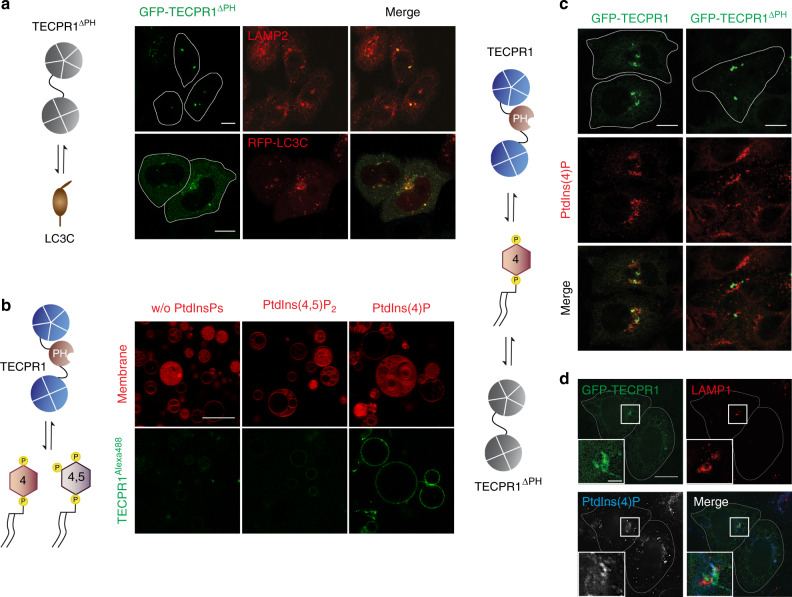
TECPR1 selectively binds PtdIns(4)P. **a** Colocalization of GFP-TECPR1^ΔPH^ with RFP-LC3C or immunostained LAMP2. Scale bars, 10 µm. **b** Binding of Alexa488-labeled TECPR1 to GUVs containing indicated PtdInsP. Scale bars, 20 µm. See also Supplementary Fig. 4b. **c** Colocalization of GFP-TECPR1 or GFP-TECPR1^ΔPH^ with immunostained PtdIns(4)P in TECPR1^KO^ cells. Scale bars, 10 µm. **d** Structured-illumination microscopy of TECPR1^KO^ cells expressing GFP-TECPR1 that were stained for LAMP1 and PtdIns(4)P. Scale bar, 10 µm, insets, 2.5 µm.

TECPR1 possesses in addition to TR1 a lipid binding PH domain. To reveal potential synergistic effects of both domains in targeting TECPR1 to lysosomes, we tested the cellular localization of a mutant in which the PH domain was deleted (TECPR1^∆PH^). Consistent with our finding that TR1 is sufficient for lysosomal targeting, we found that GFP-TECPR1^∆PH^ still colocalized with the lysosomal marker LAMP2. Moreover, GFP-TECPR1^∆PH^ still recruited LC3C puncta, indicating that the PH domain and TR1 have nonredundant and independent functions (Fig. 4a). We next tested, whether the PH domain targets TECPR1 to PtdIns(3)P enriched membranes using the PtdIns(3)P-sensor RFP-2xFYVE. Neither TECPR1^∆PH^ nor TECPR1^WT^ colocalized with RFP-2xFYVE (Supplementary Fig. 4a). We concluded that the PH domain does not bind PtdIns(3)P in vivo.

We next investigated the binding specificity of TECPR1 to PtdIns(3)P and PtdIns(3,5)P_2_ in vitro by incubating purified TECPR1 with PtdInsP-containing GUVs. No recruitment of TECPR1 to such GUVs was observed (Supplementary Fig. 4b) confirming our in vivo data that TECPR1 does not interact with PtdIns(3)P or PtdIns(3,5)P_2_.

Many PH domains are known to selectively interact with PtdIns(4,5)P_2_^19^. We thus incubated purified TECPR1 with GUVs that contained PtdIns(4,5)P_2_. Again, no interaction of TECPR1 with such GUVs was observed (Fig. 4b). Moreover, GFP-TECPR1 and GFP-TECPR1^∆PH^ did not colocalize with PtdIns(4,5)P_2_ in HeLa cells (Supplementary Fig. 4c). We continued to search for ligands of the PH domain by exploring binding of PtdIns(4)P, which is known to coordinate the fusion of autophagosomes with lysosomes^20^, to TECPR1. Interestingly, recombinant TECPR1 strongly and selectively interacted with PtdIns(4)P containing GUVs (Fig. 4b). Moreover, TECPR1 was recruited to PtdIns(4)P-positive membranes in vivo (Fig. 4c), whereas the colocalization of TECPR1^∆PH^ with PtdIns(4)P was significantly reduced (Fig. 4c and Supplementary Fig. 4d). Furthermore, structured-illumination microscopy revealed that TECPR1 colocalized with PtdIns(4)P at LAMP1-positive structures (Fig. 4d). This suggests that the PH domain targets TECPR1 to a PtdIns(4)P containing subpopulation of lysosomes, whereas lysosomal localization per se depends on the TR1 domain.

To test this idea, we depleted the pool of PtdIns(4)P in HeLa cells by knocking down the PtdIns(4)-kinase 2 alpha (PI4KIIα) and/or the PtdIns(5) phosphatase OCRL, both of which are known regulators of autophagy^20,21^. Consistent with our observation that the PH domain is dispensable for lysosomal localization of TECPR1, neither depletion of PI4KIIα or OCRL, nor that of both enzymes impacted on the recruitment of TECPR1 to LAMP2 compartments (Supplementary Fig. 4e). The few PtdIns(4)P structures that were observed in PI4KIIα knockdown cells did not colocalize with TECPR1 (Supplementary Fig. 4f). We next depleted PtdIns(4)P by overexpressing the RFP-tagged PtdIns(4)-phosphatase Sac2 and investigated whether TECPR1 still recruits LC3C. We observed a strong colocalization of TECPR1, Sac2 and LC3C, demonstrating that binding of PtdIns(4)P and recruitment of LC3C are independent functions of TECPR1 (Supplementary Fig. 4g).

In summary, we found that the recruitment of TECPR1 to lysosomes mainly depends on TR1, whereas the PH domain has a minor role.

### TECPR1 defines the subcellular destination of LC3C autophagosomes

**Fig. 5:**
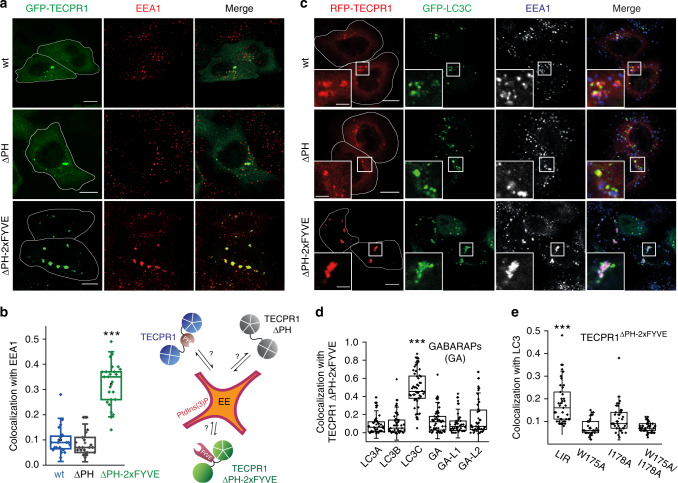
TECPR1 determines the subcellular destination of LC3C autophagosomes. **a** Colocalization of GFP-TECPR1, GFP-TECPR1^ΔPH^ or GFP-TECPR1^ΔPH-2xFYVE^ with immunostained EEA1 in TECPR1^KO^ cells. Scale bars, 10 µm. **b** Quantification of colocalization as shown in **a**, *n* = 20 cells pooled from three independent experiments. Box plots represent the first (25%) and third (75%) quartiles, respectively. The center line represents the median, whiskers the standard deviation and minima and maxima are available from the Source Data file. *P*-values were calculated using a two-tailed Student’s *t*-test (***P < 0.001). **c** Confocal images of starved TECPR1^KO^ cells coexpressing GFP-LC3C and either RFP-TECPR1, RFP-TECPR1^ΔPH^ or RFP-TECPR1^ΔPH-2xFYVE^ that were immunostained for EEA1. Scale bars, 10 µm, insets 2.5 µm. **d** Quantification of colocalization between GFP-hATG8s and RFP-TECPR1^ΔPH-2xFYVE^ as shown in Supplementary Fig. 5e. *n* = 40 cells pooled from three independent experiments. Box plots represent the first (25%) and third (75%) quartiles, respectively. The center line represents the median, whiskers the standard deviation and minima and maxima are available from the Source Data file. *P*-values were calculated using a two-tailed Student’s *t*-test (***P < 0.001). **e** Quantification of colocalization between GFP-TECPR1^ΔPH-2xFYVE^ or its LIR mutants with LC3 as shown in Supplementary Fig. 5f, *n* = 32 cells pooled from three independent experiments. Box plots represent the first (25%) and third (75%) quartiles, respectively. The center line represents the median, whiskers the standard deviation and minima and maxima are available from the Source Data file. *P*-values were calculated using a two-tailed Student’s *t*-test (***P < 0.001). See also Supplementary Movie 5. Source data are provided as a Source Data file.

Our data suggest that PtdIns(4)P regulates fusion of autophagosomes and lysosomes by targeting TECPR1 to specialized lysosomal compartments. However, PtdIns(4)P is also present in autophagic membranes^20^. To reveal closer insights into the relationship between recruitment of autophagosomes, their fusion with lysosomes and PtdIns(4)P, we uncoupled TECPR1 from autophagic and lysosomal PtdIns(4)P pools. We therefore ectopically targeted TECPR1 to endosomes by exchanging its PH against the PtdIns(3)P binding tandem-FYVE domain (TECPR1^∆PH-2xFYVE^). We first compared the colocalization of GFP-TECPR1^WT^, GFP-TECPR1^∆PH^ and GFP-TECPR1^∆PH-2xFYVE^ with the early endosomal marker EEA1. As expected, neither GFP-TECPR1^WT^ nor GFP-TECPR1^∆PH^ colocalized with EEA1, whereas a strong colocalization of GFP-TECPR1^∆PH-2xFYVE^ with EEA1 was observed (Fig. 5a, b). Interestingly, aberrant EEA1-positive structures were observed in GFP-TECPR1^∆PH-2xFYVE^-expressing cells, whereas EEA1 formed many small and dispersed puncta in GFP-TECPR1^WT^ cells. Furthermore, these EEA1 clusters colocalized with the early endosomal marker Rab5 and with late endosome/lysosome markers Rab7 and LAMP2 (Supplementary Fig. 5a). Moreover CLEM experiments revealed that GFP-TECPR1^∆PH-2xFYVE^ colocalized with multivesicular bodies (MVBs, Supplementary Fig. 5b), suggesting that TECPR1^∆PH-2xFYVE^ induces the formation of atypical endosomes. To exclude that endosomal trafficking or degradation of endocytosed material is perturbed in cells expressing TECPR1^∆PH-2xFYVE^, we next performed epidermal growth factor receptor (EGFR) degradation assays. No difference in the decline of EGFR levels was observed in TECPR1^KO^ cells (Supplementary Fig. 5c) or in cells expressing TECPR1^∆PH^ or TECPR1^∆PH-2xFYVE^ (Supplementary Fig. 5d). This shows that TECPR1 is dispensable for EGFR degradation and that clustering of atypical endosomes in TECPR1^∆PH-2xFYVE^-expressing cells does not impair endosomal trafficking.

Many lysosomal tethering factors contribute to the recruitment of autophagosomes to lysosomes. To determine whether TECPR1 acts independently of these factors, we analyzed the recruitment of GFP-LC3C puncta to early endosomes in TECPR1^KO^ cells that expressed RFP-TECPR1^WT^, -TECPR1^∆PH^ or -TECPR1^∆PH-2xFYVE^. Small EEA1 puncta that did not colocalize with LC3C were observed in TECPR1^WT^ or TECPR1^∆PH^-expressing cells. Similar LC3C-negative EEA1 puncta were also present in cells expressing TECPR1^∆PH-2xFYVE^. However, these cells contained a second population of bigger EEA1 clusters at which LC3C and TECPR1^∆PH-2xFYVE^ colocalized (Fig. 5c and Supplementary Movie 5). This observation demonstrates that TECPR1 recruits LC3 autophagosomes independently of PtdIns(4)P or of other lysosomal proteins.

Given that TECPR1 selectively interacts with LC3C, we predicted that other ATG8s are not rerouted to endosomes. To test this prediction, we analyzed colocalization of all ATG8s with EEA1 in TECPR1^∆PH-2xFYVE^-expressing cells. We found that only GFP-LC3C strongly colocalized with TECPR1^∆PH-2xFYVE^ at EEA1 clusters, whereas no or very weak colocalization was observed for other hATG8s (Fig. 5d and Supplementary Fig. 5e). This confirms that TECPR1 selectively recruits LC3C vesicles.

Our data showed that the LIR motif of TECPR1 is important, yet not essential for LC3C binding. We therefore tested whether LC3C vesicles were rerouted to endosomes if the LIR motif in TECPR1^∆PH-2xFYVE^ was inactivated in LIR mutants W175A, I178A and W175A/I178A. We found that these LIR mutants were still recruited to and induced clustering of EEA1 (Supplementary Fig. 5f). However, a significantly reduced colocalization of these mutants with endogenous LC3 was observed (Fig. 5e and Supplementary Fig. 5f). Thus, the LIR motif of TECPR1 is not only important for the interaction of TECPR1 with hATG8s in vitro, it also facilitates the recruitment of LC3 in vivo. Taken together, we demonstrate here that the localization of TECPR1 defines the intracellular destination of LC3C vesicles independently of other co-factors. This suggests that TECPR1 acts upstream of other lysosomal proteins that have previously been shown to coordinate autophagosome–lysosome fusion such as the HOPS complex^22,23^.

### TECPR1 and LC3C coordinate targeting of protein aggregates to lysosomes

**Fig. 6:**
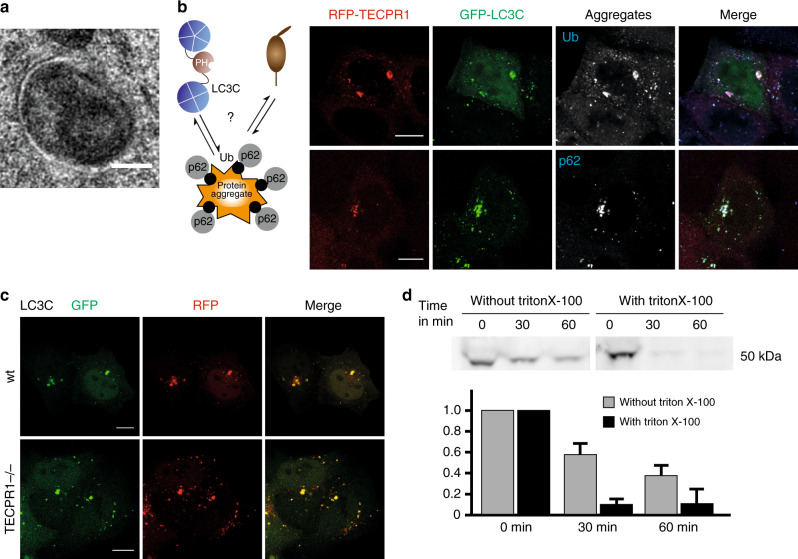
TECPR1 and LC3C coordinate targeting of aggregates to lysosomes. **a** Correlative light electron micrograph of HeLa cells that expressed RFP-LC3C. The electron micrograph shows a 70 nm section. RFP-LC3C fluorescence correlated with a double membrane structure that surrounds electron-dense amorphous material. Scale bar, 200 nm. **b** Confocal images of puromycin-treated TECPR1^KO^ cells that co-expressed RFP-TECPR1 and GFP-LC3C and that were immunostained for Ub or p62. Scale bars, 10 µm. **c** Confocal images of puromycin-treated wild-type (wt) and TECPR1^KO^ cells expressing RFP-GFP-LC3C. Most structures displayed GFP and RFP fluorescence. Scale bars, 10 µm. **d** Proteinase K protection assay of GFP-LC3C transfected HeLa cells. Immunoblotting was performed using an anti-GFP antibody. Lysates were incubated with Proteinase K for indicted times in the absence or presence of Triton X-100. The chart shows quantification of three independent experiments (mean ± SD). Source data are provided as a Source Data file.

A number of recent studies demonstrated that LC3C has multiple functions in autophagy^24–27^. Not surprisingly, the number of LC3C puncta exceeds by far that of the other ATG8 homologs (Fig. 1c). In order to characterize the pool of LC3C autophagosomes that is selectively recognized by TECPR1, we made use of the ectopic targeted TECPR1^∆PH-2xFYVE^ construct. We first asked whether TECPR1 recruits LC3C vesicles that contain a specific type of cargo by determining the colocalization of GFP-LC3C puncta with selective cargo markers in RFP-TECPR1^∆PH-2xFYVE^-expressing cells. The two organellar markers Hsp60 (mitochondrial chaperonin) and PMP70 (peroxisomal membrane protein) did not colocalize with LC3C (Supplementary Fig. 6a). However, LC3C strongly colocalized with the autophagy receptor p62 and with ubiquitin (Ub) (Supplementary Fig. 6b), both of which are implemented in many types of selective autophagy. To reveal deeper insights into the nature of cargo transported by LC3C vesicles, we characterized the ultrastructure of these vesicles by CLEM. We found that RFP-LC3C fluorescence colocalized with autophagosomes that contained electron-dense, amorphous material that resembles protein aggregates (Fig. 6a). To verify that LC3C is involved in aggrephagy, we next selectively increased the number of protein aggregates by treating HeLa cells with puromycin. We found that the vast majority of Ub/p62-positive puncta colocalized with GFP-LC3C (Fig. 6b), arguing that LC3C vesicles are indeed autophagosomes that contain protein aggregates.

The major function of p62 is to tether cargo to the inner autophagic membrane through its interaction with hATG8s that are conjugated to the inner membrane of autophagosomes. This confines p62 and hATG8s in the lumen of autophagosomes, which leads to their degradation in lysosomes. Moreover, luminal hATG8s are inaccessible for lysosomal TECPR1. To reveal the primary localization of LC3C on autophagosomes, we used the pH-sensitive RFP-GFP-LC3C reporter. We found that the majority of LC3C puncta in wild-type as well as TECPR1^KO^ cells was yellow (Fig. 6c), suggesting that LC3C is mainly conjugated to the outer autophagic membrane. To confirm our findings, we next performed proteinase K protection assays of lysates of GFP-LC3C-expressing cells to determine the fraction of LC3C that is accessible by the protease (Fig. 6d). We found that ~60% of GFP-LC3C was degraded after 60 min of proteinase K treatment in the absence, and 90% in the presence of detergent. This shows that the majority of LC3C is not protected from proteinase K and thus likely to be conjugated to the cytoplasmic leaflet of autophagosomes. The smaller fraction of GFP-LC3C (~30%) that was protected from proteinase K resides within autophagosomes, which is consistent with the previously reported function of LC3C to be a cargo tether.

To consolidate our model that TECPR1 and LC3C cooperate to promote aggrephagy, we next performed a comprehensive series of pulse-chase experiments to follow clearance of puromycin-induced protein aggregates. We first depleted LC3C from wild-type or TECPR1^KO^ cells by siRNA (Supplementary Fig. 6d) followed by puromycin treatment for 2–4 h (pulse). We then chased the clearance of protein aggregates in the presence and absence of the proteasome inhibitor MG132. We observed that the clearance of protein aggregates was not impaired in TECPR1^KO^ cells that expressed normal levels of LC3C. Moreover, additional depletion of LC3C in TECPR1^KO^ cells induced only a mild accumulation of aggregates (Supplementary Fig. 6d, e), suggesting that TECPR1 and LC3C are not essential. To test whether other ATG8 proteins are involved in this pathway, we next depleted either all LC3 proteins or all GABARAP proteins in puromycin-treated wild-type cells, followed by puromycin retrieval. Again, a moderate delay in protein clearance was observed in cells lacking LC3s, whereas knockdown of all GABARAP proteins had a much stronger effect (Supplementary Fig. 6e, f). This observation is consistent with several other studies that reported a stronger impairment of autophagy upon depletion or deletion of GABARAPs compared to that of LC3s^28,29^. The greater dependency on GABARAPs was attributed to their function in autophagosome–lysosomes fusion. We predicted that depletion of GABARAPs leads to an accumulation of LC3 puncta. To test this prediction, we treated TECPR1^∆PH-2xFYVE^-expressing cells with siRNAs against all GABARAPs and investigated clustering of LC3 puncta at EEA1-positive compartments (Supplementary Fig. 6g). We indeed observed a significantly enhanced colocalization of LC3, EEA1 and TECPR1^∆PH-2xFYVE^ (Supplementary Fig. 6g, h), suggesting that GABARAPs are dispensable for LC3 puncta formation but required for lysosomal clearance of protein aggregates.

### TECPR1 promotes aggregate clearance in neural cells

**Fig. 7:**
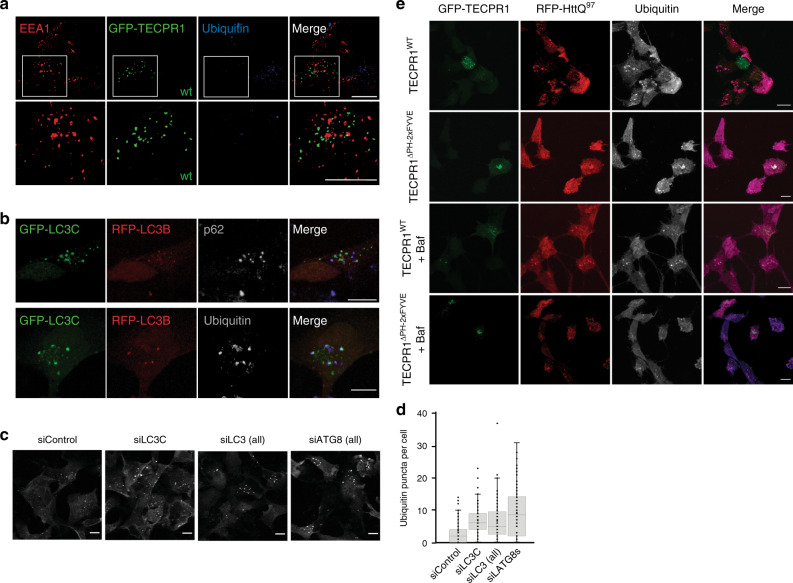
TECPR1 promotes aggregate clearance in neural cells. **a** Confocal images of neural stem cells expressing GFP-TECPR1 that were immunostained for EEA1 and Ubiquitin. The lower panel shows a magnification of the area that is indicated in the upper panel. Scale bar = 10 µm. **b** Confocal images of NSCs coexpressing GFP-LC3C and RFP-LC3B, costained for p62 or ubiquitin as indicated. Scale bar = 10 µm. **c** Confocal images of NSCs that were treated with siRNAs against ATG8 proteins as indicated and immunostained for ubiquitin. Scale bars, 10 µm. **d** Quantification of Ub puncta per cells from data corresponding to representative images as shown in **c** from cells of randomly chosen field of views (*n* = 70 cells pooled from four independent experiments). Box plots represent the first (25%) and third (75%) quartiles, respectively. The center line represents the median, whiskers the standard deviation and minima and maxima are available from the Source Data file. **e** Confocal images of NSCs expressing RFP-HttQ^97^ and GFP-TECPR1 or GFP-TECPR1^∆PH2xFYVE^, immunostained for ubiquitin were treated with the lysosomal inhibitor Bafilomycin A1 (Baf) as indicated. Note that for merged images, ubiquitin is shown in the blue channel instead of gray. Scale bar = 10 µm. See also Supplementary Movie 6. Source data are provided as a Source Data file.

The hallmark of neurodegenerative diseases is the accumulation of Ub- and p62-positive protein aggregates in neural cells. We thus tested whether LC3C and TECPR1 are essential for aggrephagy in neural stem cells that expressed GFP-TECPR1 or its variant GFP-TECPR1^∆PH-2xFYVE^. Consistent with our findings in HeLa cells, TECPR1^WT^ colocalized with LAMP1, whereas TECPR1^∆PH-2xFYVE^ predominantly associated with EEA1-positive structures in NSCs (Supplementary Fig. 7a, b). We next analyzed the number and morphology of Ub puncta in wild-type and GFP-TECPR1-expressing cells. As expected, small Ub puncta were detected in wild-type NSCs. However, almost no Ub puncta were observed in NSCs that expressed GFP-TECPR1 (Fig. 7a), suggesting that TECPR1 promotes clearance of protein aggregates. Furthermore, TECPR1^∆PH-2xFYVE^ transfected NSCs contained fewer but much larger Ub puncta, most of which colocalized with EEA1 (Supplementary Fig. 7c and d). Thus, expressing TECPR1^∆PH-2xFYVE^ in NSCs induces the formation of aberrant EEA1 structures at which Ub clusters.

We next investigated whether the observed decline in Ub puncta upon overexpression of TECPR1^WT^ depends on autophagy using the autophagy-inhibitor Bafilomycin A1 (BafA1). We found that under these conditions also cells that expressed TECPR1^WT^ contained Ub puncta (Supplementary Fig. 7e). Similar effects were not observed in HeLa cells, providing further evidence that neural cells strongly depend on TECPR1 to maintain their protein homeostasis. We next treated NSCs with puromycin to selectively increase the number of protein aggregates. We found that even under these conditions, GFP-TECPR1-expressing NSCs were devoid of Ub puncta, whereas Ub puncta were observed when autophagy was inhibited by BafA1 (Supplementary Fig. 7f, g). Collectively, these results show that TECPR1 promotes the clearance of protein aggregates in NSCs by augmenting autophagy.

Our studies in HeLa cells revealed a close collaboration of LC3C and TECPR1 in aggrephagy. Consistent with this observation, Ub puncta colocalized with LC3C in puromycin-treated NSCs and in BafA1-treated NSCs that expressed GFP-TECPR1 (Supplementary Fig. 7f). Moreover, Ub and LC3C-positive puncta also colocalized with the autophagy receptor p62, whereas no significant colocalization with RFP-LC3B was detected, confirming the selective recruitment of LC3C to such puncta (Fig. 7b and Supplementary Movie 6). To confirm that the formation of LC3C puncta was autophagy-dependent, we next inhibited autophagy by treating NSCs with the PI3-kinase inhibitor wortmannin. We found that the number of LC3C puncta was strongly reduced while puncta formation of TECPR1 was not disturbed (Supplementary Fig. 7h). Moreover, TECPR1 puncta still colocalized with lysosomal markers. Wortmannin inhibits not only autophagy but also endosomal pathways. We thus treated GFP-TECPR1-expressing NSCs with the selective VPS34 inhibitor SAR405 and investigated colocalization of TECPR1 with LAMP1 and lysotracker. We found that most TECPR1 puncta did not colocalize with both lysosomal markers, suggesting that the recruitment of TECPR1 to lysosomes depends on autophagy (Supplementary Fig. 7i). Such an interdependence between TECPR1 localization and autophagy is consistent with our observation that TECPR1 preferentially associates with autolysosomes instead of lysosomes (Supplementary Fig. 1e).

To explore the recruitment of LC3C vesicles by TECPR1 in more detail, we followed trafficking of LC3C puncta to TECPR1 structures in puromycin-treated NSCs that co-expressed RFP-LC3C and GFP-TECPR1 or GFP-TECPR1^∆PH-2xFYVE^. We observed that RFP-positive TECPR1 compartments fused with several LC3C puncta during the imaging time in cells expressing GFP-TECPR1 (Supplementary Movies 7 and 8). Moreover, expression of GFP-TECPR1^∆PH-2xFYVE^-induced clustering of LC3C puncta, which did not fuse with TECPR1^∆PH-2xFYVE^ structures (Supplementary Movies 9 and 10). Taken together, we found that TECPR1 is preferentially recruited to autolysosomes that fuse with several LC3C vesicles.

We next investigated whether fusion of LC3C and TECPR1 compartments correlated with delivery of protein aggregates to lysosomes using GFP-RFP-p62 as reporter. The confinement of p62 in the lumen of autophagosomes leads to its delivery to lysosomes, where the fluorescence of GFP but not that of RFP is quenched. We found that more than 90% of p62 puncta are yellow in wild-type NSCs, whereas a significant increase in the fraction of red p62 puncta was observed in cells expressing GFP-TECPR1. Moreover, TECPR1^∆PH-2xFYVE^ transfected cells contained mostly yellow p62 puncta (Supplementary Fig. 7k). Taken together, significantly more p62 is delivered to lysosomes in TECPR1-expressing cells, suggesting that overexpression of TECPR1 leads to an increased flux of autophagic cargo in NSCs.

Given that TECPR1 selectively interacts with LC3C, we next explored the contribution of LC3C to aggrephagy by depleting either LC3C alone, all three LC3s or all six hATG8s collectively from NSCs using siRNA. We found that Ub puncta strongly accumulated in LC3C depleted cells (Fig. 7c, d), whereas no further increase in the number or size of Ub clusters was observed in cells depleted for all LC3s. This observation confirms that LC3A and LC3B are not significantly contributing to aggrephagy. However, siRNA of all hATG8s together resulted in a stronger accumulation of Ub puncta (Fig. 7c, d), demonstrating that LC3C and GABARAPs act independently with LC3C promoting recruitment of autophagosomes, whereas GABARAPs facilitate their fusion with lysosomes.

Collectively, our study provides strong evidence that TECPR1 and LC3C promote clearance of protein aggregates in NSCs. This raises the question whether the pathway is also required to degrade disease-related protein aggregates in neural cells. To answer this question, we co-expressed RFP-tagged poly-glutamine Huntingtin (HttQ^97^) with TECPR1 or TECPR1^∆PH2xFYVE^ in NSCs. We found that cells expressing TECPR1 were devoid of RFP-HttQ^97^ and Ub puncta, whereas many puncta were present in non-transfected cells (Fig. 7e). Moreover, bigger HttQ^97^ puncta that colocalized with Ub were detected in TECPR1^∆PH2xFYVE^-expressing NSCs. Finally, Htt aggregates were present in TECPR1 and TECPR1^∆PH2xFYVE^-expressing cells if autophagy was inhibited by BafA1 treatment (Fig. 7e), demonstrating that the clearance of Htt aggregates occurs in an autophagy-dependent fashion.

Taken together, our study provides compelling evidence that TECPR1 and LC3C are key components of a specialized pathway that promotes aggrephagy in neural cells.

## Discussion

Selective autophagy is a pivotal recycling mechanism in eukaryotic cells that ensures cellular homeostasis to be maintained. The selection of cargo, including damaged or superfluous organelles but also protein aggregates, is coordinated by a set of autophagy receptors. The major function of these receptors is to tether cargo to autophagic membranes by binding hATG8s that are conjugated to the inner membrane of autophagosomes^25,27,30^. A second pool of hATG8s is conjugated to the outer membrane of autophagosomes with LC3B being the most abundant and best characterized family member during starvation-induced autophagy^30^.

Here we show that the majority of LC3C structures contain protein aggregates, which also colocalize with STX17. Moreover, we found that most of LC3C is not reaching the acidic lumen of lysosomes, suggesting that LC3C is conjugated to the outer membrane of autophagosomes. Furthermore, lysosomal TECPR1 selectively recruits LC3C vesicles based on a physical interaction between both proteins. This interaction depends on the canonical LIR W^175^AKI^177^ and the TR1 domain of TECPR1. Finally, we show that TECPR1 promotes the fusion of LC3C autophagosomes with lysosomes to promote degradation of protein aggregates.

The fusion of autophagosomes and lysosomes requires a complex molecular machinery that coordinates the specific recruitment, tethering and fusion of both organelles. Not surprisingly, many factors are known to coordinate this process including the tethering factors EPG5, HOPS and PLEKHM1^22,23,31^ as well as SNARE proteins STX17, SNAP29 and VAMP8^32,33^. In order to untangle TECPR1 functions from the apparently complex interaction network of other lysosomal proteins, we targeted TECPR1 to endosomal compartments by exchanging its PH domain against a 2xFYVE domain. We found that TECPR1^∆PH-2xFYVE^ reroutes LC3C from lysosomes to endosomes, demonstrating that TECPR1 is a lysosomal recruitment factor that acts independently and most upstream in a series of events that leads to the fusion of LC3C autophagosomes with lysosomes.

Moreover, we characterized the binding specificity and function of the PH domain of TECPR1 to reveal its contribution to aggrephagy. We found that the PH domain selectively binds PtdIns(4)P in vitro and is required to target TECPR1 to PtdIns(4)P-positive lysosomes in vivo. This observation is significant because increasing experimental evidence revealed that PtdIns(4)P regulates fusion of autophagosomes and lysosomes^20,34^. Our study revealed a yet unknown function of lysosomal PtdIns(4)P. We showed that the PH domain recruits TECPR1 to a population of lysosomes that contains PtdIns(4)P. Moreover, we found that TECPR1 is preferentially recruited to autolysosomes, which fuse with several LC3C autophagosomes. Collectively, these observations imply that a subpopulation of TECPR1 and PtdIns(4)P-positive lysosomes represent specialized compartments to degrade autophagic cargo.

Interestingly, an accumulation of TDP-43 aggregates in frontotemporal dementia and amyotrophic lateral sclerosis correlates with a significant downregulation of TECPR1 expression in neurons^14,15^. Moreover, autophagosomes accumulate in neurons of patients who suffer from other neurodegenerative diseases including Huntington’s, Alzheimer’s and Parkinson’s disease. This suggests that protein homeostasis in neurons depends strongly on autophagy in general and TECPR1 in particular. We found that overexpression of TECPR1^WT^ in NSCs facilitates clearance of protein aggregates in an autophagy-dependent manner. Moreover, targeting TECPR1 to endosomal compartments or inhibition of autophagy leads to a strong accumulation of LC3C autophagosomes and of HttQ^97^ inclusions. Correspondingly, an accumulation of protein aggregates is also observed in NSC in which LC3C has been depleted. The neuroprotective function of TECPR1 that we discovered in our study implies that its augmented expression could potentially counteract the onset of neurodegenerative diseases.

## Methods

### Reagents

The following synthetic lipids were purchased from Avanti Polar Lipids: 1-palmitoyl-2-oleoyl-*sn*-glycero-3-phosphocholine (POPC), 1,2-dioleoyl-*sn*-glycero-3-phosphocholine (DOPC), 1-palmitoyl-2-oleoyl-*sn*-glycero-3-phosphoserine (POPS), 1,2-dioleoyl-*sn*-glycero-3-phosphoethanolamine (DOPE), cholesterol, lissamine-rhodamine-PE, 1,2-dioleoyl-*sn*-glycero-3-phosphoinositol-3′-phosphate (PtdIns(3)P), 1,2-dioleoyl-*sn*-glycero-3-phosphoinositol-3′,5′-bisphosphate (PtdIns(3,5)P_2_) and 1,2-dioleoyl-*sn*-glycero-3-phosphoinositol-4′-phosphate (PtdIns(4)P). PI3-kinase inhibitor Wortmannin was purchased from Sigma (W1628) and the VPS34-specific inhibitor SAR405 from Clinisciences (A8883-1ml).

### Antibodies

The following antibodies were used in this study: anti-HA (Santa Cruz, sc-7392), anti-β-actin (Invitrogen, MA1-140), anti-LAMP2 (Santa Cruz, sc-18822), anti-LAMP1 (CST, 9091), anti-LC3 (MBL International, M152-3), anti-p62 (BD Biosciences, 610833), anti-STX17 (GeneTex, GTX130212), anti-EEA1 (CST, 3288), anti-Rab5 (CST, 3547), anti-Rab7 (CST, 9367), anti-Rab11A (Invitrogen, 700184), anti-Ub (Enzo Life Sciences, BML-PW8810), anti-Hsp60 (CST, 12165), anti-PMP70 (Sigma-Aldrich, SAB4200181), anti-EGFR (Santa Cruz, sc-03-G), anti-PtdIns(4)P (Echelon, Z-P004), anti-GFP (Invitrogen, A-6455), anti-myc (9E10, Biochemistry core facility, MPI of Biochemistry, Martinsried), anti-PtdIns(4,5)P_2_ (Echelon, Z-P045) and anti-HA (CST, 3724).

### Plasmids

Full-length cDNAs encoding isoform 1 of human LC3A, LC3B, LC3C, GABARAP, GABARAPL1, GABARAPL2, ATG3, ATG5, ATG7, ATG10, ATG12 and TECPR1, and HGS were amplified by PCR from cDNA libraries (imaGenes GmbH). For recombinant expression of proteins, cDNAs were cloned into pCoofy vectors^28^. For expression of GFP-tagged hATG8 proteins in mammalian cells, cDNAs were inserted into pEGFP-C1 between *Bgl*II and *Kpn*I restriction sites. TECPR1 and the TECPR1 domains TR1 (aa 1–377) and TR2 (aa 722–1165) were cloned into pEGFP-C1 between *Xho*I and *Hin*dIII restriction sites, resulting in GFP-TECPR1, GFP-TR1 and GFP-TR2. The plasmid ptfLC3 encoding mRFP-eGFP-LC3 was a gift from Tamotsu Yoshimori (Addgene plasmid # 21074). To generate RFP-GFP-LC3C, LC3 was replaced by LC3C by inserting LC3C cDNA into pTfLC3 between *Bgl*II and *Kpn*I restriction sites. N-terminal HA-tagged LC3 proteins were generated by cloning LC3A, LC3B and LC3C cDNAs together with 3xHA separated by a AQCS(GA)_6_GPTENSS linker into pLPCX (Clontech) between *Hin*dIII and *Not*I restriction sites. The plasmid pmRFP-C1 was generated by substitution of eGFP in pEGFP-C1 with mRFP from pTfLC3 using *Nhe*I and *Bsp*EI/*Age*I restriction enzymes. LC3C and TECPR1 constructs were inserted into pmRFP-C1 to generate the N-terminal RFP-tagged proteins RFP-LC3C and RFP-TECPR1. Deletion of the PH domain in TECPR1 (TECPR1^ΔPH^, Δaa 611–717) was introduced by PCR linearization of GFP-TECPR1 or RFP-TECPR1, followed by homologous recombination using RecAf (NEB). TECPR1^ΔPH-2xFYVE^ was generated by replacing the PH domain (aa 611–717) in GFP-TECPR1 and RFP-TECPR1 by two repeats of the FYVE domain of HGS (aa 147–222) separated by a QGQGS linker. TECPR1 constructs (TECPR1^wt^, TECPR1^ΔPH^, TECPR1^ΔPH-2xFYVE^) were cloned into pLPCX between *Hind*III and *Not*I restriction sites. To construct the PtdIns(3)P-sensor RFP-2xFYVE, two repeats of the FYVE domain of HGS (aa 147–222) separated by a QGQGS linker were cloned into pmRFP-C1 between *Xho*I and *Bam*HI restriction sites. MycBioID-TECPR1 was cloned by amplification of BioID2 from MCS-BioID2-HA, which was a gift from Kyle Roux (Addgene plasmid # 74224) and subsequent insertion together with an N-terminal Myc-tag into TECPR1 in pLPCX between XhoI and HindIII restriction sites. TECPR1 LIR mutants (W175A, I178A, W175A/I178A) were generated using the QuikChange Lightning Site-Directed Mutagenesis Kit (Agilent) according to the manufacturer’s instructions. The plasmid pRC containing the aggregate prone variant HttQ97 was a gift from F. Ulrich Hartl^35^.

### Primers

Primers used for cloning of hATG8s. Gene-specific sequences are black, restriction sites are marked in blue, homolog sequences to the linearized vector are marked in green, and additionally added base pairs are indicated in red.



Primers used for cloning of TECPR1 and TECPR1 constructs. Gene-specific sequences are black, restriction sites are marked in blue, homolog sequences to either the linearized vector or the preceding ORF sequence are marked in green, and additionally added or altered base pairs are marked in red.



Primers used for cloning of RFP-2xFYVE. Gene-specific sequences are black, homolog sequences to either the linearized vector or the preceding ORF sequence are marked in green, and additionally added base pairs are marked in red.

Guide RNAs used for CRISPR/Cas9-mediated gene knockout of TECPR1 and ATG16L1. Added *Bbs*I restriction sites are indicated in blue and additionally added G–C pairs are indicated in red.



### Expression and purification of proteins

ATG3 and the hATG8 proteins LC3A, LC3B, LC3C, GABARAP, GABARAPL1 and GABARAPL2 were cloned into pCoofy1, resulting in N-terminal His_6_-tagged proteins. hATG8 proteins were expressed with a C-terminal deletion that reveals the reactive glycine and with an N-terminal cysteine for fluorescent labeling. The ATG12–ATG5 conjugate was produced by coexpressing ATG7, ATG10, ATG12 and ATG5 from the polycystronic vector pST39^36^ with a His-tag fused to the N terminus of ATG12. The TECPR1 peptide (TECPR1 158-187) and corresponding W175A/I178A LIR mutant were cloned into pCoofy4 and expressed with N-terminal His_6_-MBP-tag. ATG3, hATG8s, ATG12–ATG5 and TECPR1 peptides were expressed in *E. coli* Rosetta. Cultures were grown in LB-medium and induced with 0.3 mM IPTG for 18 h at 18 °C. Cells were harvested and resuspended in lysing buffer (100 mM Tris-HCl pH = 8.0, 300 mM NaCl, 20 mM imidazole, 10% glycerol) supplemented with protease inhibitor cocktail (Sigma-Aldrich, P8849) as well as Benzonase (Sigma-Aldrich). *E. coli* cells were lysed by sonication for 20 min at 4 °C (Sonopuls, Bandelin). ATG7 was cloned into pCoofy27 to express N-terminal His_6_-tagged protein and TECPR1 was cloned into pCoofy29, resulting in N-terminal His_6_-MBP-tagged proteins. ATG7 was expressed in SF9 and TECPR1 in High Five insect cells for 72 h at 25 °C^37^. Insect cells were harvested and resuspended in lysing buffer supplemented with protease inhibitor cocktail as well as Benzonase. Insect cells were lysed using a dounce homogenizer. Both *E. coli* and insect cell lysates were centrifuged at 45,000×*g* for 1 h at 4 °C and the supernatant was incubated with 1 ml Ni-NTA agarose (QIAGEN) for 1 h at 4 °C. Subsequently, the affinity resin was washed and eluted with elution buffer (50 mM Tris-HCl pH = 7.4, 300 mM NaCl, 500 mM imidazole, 10% glycerol). The affinity tags were cleaved by PreScission protease digest and digested proteins were subjected to size-exclusion chromatography on a Superdex 75 (hATG8 proteins and ATG3) or Superdex 200 column (all others) using 25 mM Tris-HCl pH 7.4, 275 mM NaCl as running buffer. TECPR1 peptides were directly subjected to a Superdex 200 column without cleavage of the His_6_-MBP-tag. Fractions containing target protein were pooled, concentrated, aliquoted, flash frozen in liquid nitrogen and stored at −80 °C until use.

### Fluorescent labeling of proteins

LC3C and TECPR1 were labeled by coupling Alexa Fluor 488 C_5_ Maleimide (Molecular Probes) or CF®405 M (Biotium) to the introduced N-terminal cysteines or to native cysteines, respectively. Therefore, proteins were mixed with fluorescent dye in a 1:1 ratio, incubated for 1 h at room temperature, and unbound dye was removed using a HiTrap Desalting Column (GE Healthcare).

### Floatation assay

For generation of small unilamellar vesicles (SUVs), dried lipids were dissolved in reaction buffer (12.5 mM Tris-HCl pH = 7.4, 137.5 mM NaCl, 0.1 mM DTT, 1 mM ATP/Mg^2+^), subjected to three freeze-thaw cycles and sonicated until the solution was clear. Lipid mixtures contained 59.9 mol% DOPC, 40 mol% DOPE, and 0.1 mol% lissamine-rhodamine-PE. Final protein stoichiometries for lipidation reactions were ATG7:ATG3:hATG8:ATG12–ATG5, 1:1.5:6:0.5, respectively. SUVs were incubated with the protein-mix in reaction buffer for 1 h at 37 °C. The protein/liposome mix was mixed with one volume 80% Histodenz in floatation buffer (25 mM HEPES pH = 7.0, 100 mM NaCl) and overlaid with 30% Histodenz and floatation buffer to generate a Histodenz step gradient (40%/30%/0%). After ultracentrifugation at 165,000×*g* for 1 h, floated fractions containing proteoliposomes (liposome fraction) and unbound proteins (protein fraction) were analyzed by SDS-PAGE using NuPAGE 4–12% Bis-Tris gels (Novex).

### PtdInsP binding assay

Giant unilamellar vesicles (GUVs) were prepared by electroformation^38^ using lipid mixtures containing POPC (36.9 mol%), DOPE (30 mol%), cholesterol (20 mol%), POPS (10 mol%), PtdInsPs (3 mol%) and lissamine-rhodamine-PE (0.1 mol%). PtdInsPs were protonated before incorporation into GUVs. 100 µl GUVs were incubated with 100 µl of 1 µM TECPR1 in binding buffer (25 mM Tris-HCl pH = 7.4, 275 mM NaCl, 0.2 mM DTT) for 30 min at 37 °C in an observation chamber (Lab-Tek).

### Cell culture and transfection

HeLa cells were purchased from Cell Lines Service (# 300194). HeLa cells were cultured in DMEM (Gibco, 31966-021) supplemented with 10% FBS (Sigma-Aldrich, F4135) and 1× Penicillin–Streptomycin (Gibco, 15140-122). Human neural stem cells (NSCs), derived from H9 (WA09) human embryonic cell lines, were purchased from ThermoFisher scientific (# N7800). NSCs were cultured in StemPro media (ThermoFisher Scientific) containing basic fibroblast growth factor (FGF-2, 10 ng/ml) and Epidermal growth factor (EGF, 10 ng/ml). Culture plates and glass coverslips were coated with Geltrex (ThermoFisher scientific, 12063569). HeLa cells and NSCs were cultured at 37 °C in a 5% CO_2_ incubator. HeLa cells were starved by washing them three times with DPBS and incubation in Earle’s Balanced Salt Solution (EBSS; Sigma-Aldrich, E2888) for 2 h. For lysotracker treatment, LysoTracker Deep Red (ThermoFisher Scientific) was added to the cells in a concentration of 100 nM 30 min prior to imaging. Formation of protein aggregates was induced by treatment of cells with 5 µg/ml puromycin for 2 h. HeLa cells were transfected with TransIT-HeLaMONSTER® Transfection Kit (Mirus, MIR2904) according to the manufacturer’s instructions and analyzed 24 h post transfection. Transfection of NSCs was performed using the TransIT®-293 Transfection Reagent (Mirus, MIR2704) and samples were analyzed 24–36 h post transfection. Autophagy was inhibited by treating cells with 400 nM of the autophagy-inhibitor Bafilomycin A1 (Sigma-Aldrich, B1793) for 4 h. The PI3-kinase was inhibited using wortmannin (SIGMA, W1628) at a final concentration of 1.5 mM for 4 h or the VPS34-specific inhibitor SAR405 at a final concentration of 10 µM for 2 h. Knockdowns of ATG8 variants was performed by incubating HeLa cells or NSCs with Silencer siRNAs (Invitrogen, 4427037) at a final concentration of 10 nM for 48 h. Cotransfection with other plasmids was performed 24 h after addition of siRNAs and cells were incubated for another 24 h Table 1.

**Table 1:** siRNAs from Invitrogen.

siRNA	Order number	Sequence (5′–3′)
siControl	Stealth RNAi	CAACUUGAUCCGUCUGACGUGGAAU
siLC3A	s39156	
siLC3B	s37748	
siLC3C	Stealth RNAi	GCUUGGCAAUCAGACAAGAGGAAGU
siGABARAP	s22361	
siGABARAPL1	s24332	
siGABARAPL2	s223228	
siTECPR1	Stealth RNAi	CCAGUUGGAUUGAGAUGGUUGGUGA

### Generation of KO cell lines using CRISPR-Cas9

A HeLa KO cell line deficient for TECPR1 was generated according to^39^. In brief, CRISPR guide RNAs that target the first exon (Supplementary Table 1) were cloned into pSpCas9(BB)-2A-Puro (PX459), which was a gift from Feng Zhang (Addgene plasmid # 48139). Transfected HeLa cells were selected with puromycin and clonal cell lines were isolated by dilution. Genomic regions containing the editing site were amplified by PCR and screened for frameshift mutations by sequencing.

### BioID analysis

BioID^34^ was performed by cotransfecting HeLa TECPR1^KO^ cells with MycBioID-TECPR1, the corresponding W175A/I178A LIR mutant, or pLPCX (mock) and HA-tagged hATG8s. 50 μM biotin was added at time of transfection and cells were lysed 24 h post transfection. Lysates were incubated with Dynabeads MyOne Streptavidin C1 (Invitrogen, 65001) overnight at 4 °C. Beads were washed once with 2% SDS in 50 mM Tris-HCl pH = 7.4 and twice with BioID washing buffer (50 mM Tris-HCl pH = 7.4, 500 mM NaCl, 1% Triton X-100, 1 mM EDTA). Bound proteins were eluted in 2× SDS-PAGE sample buffer + 3 mM biotin. Samples were then blotted with anti-HA, anti-Myc and Streptavidin-HRP (ThermoScientific, 21130).

### Immunocytochemistry

Cells grown on glass coverslips were fixed for 10 min with 4% formaldehyde, washed three times with PBS, permeabilized in permeabilization solution (0.2% Triton X-100, 0.5% SDS, 4% BSA in PBS) for 5 min, washed three times with PBS and incubated in blocking solution (4% BSA in PBS) for 1 h at room temperature. Primary antibodies were diluted 1:100 in blocking solution and incubated for 1 h at room temperature. After washing with PBS, samples were incubated with Alexa Fluor secondary antibodies (Molecular Probes, dilution 1:500 in blocking solution) for 1 h at room temperature. Immunofluorescence with anti-PtdIns(4)P, anti-LC3 and anti-Rab11A was performed according to the “Golgi staining” protocol^35^. Immunofluorescence staining for PtdIns(4,5)P_2_ was performed according to the manufacturer’s instructions. Coverslips were mounted on slides with ProLong Gold antifade reagent (Invitrogen).

### Immunoblotting

HeLa cells were lysed with mammalian lysis buffer (20 mM Tris pH = 7.5, 150 mM NaCl, 1 mM EDTA, 1 mM EGTA, 1% Triton X-100) supplemented with protease inhibitor cocktail (Sigma-Aldrich, P8340). Cell lysates were subjected to SDS-PAGE, transferred to a PVDF membrane, and blotted with primary and secondary antibodies.

### Confocal and superresolution microscopy

Confocal images were acquired on a Leica TCS SP8 AOBS Confocal Laser Scanning Microscope (×63/1.4 NA objective for GUVs and fixed cells and ×63/1.2 NA objective for live cell imaging) using the Leica LAS AF SP8 software. Structured-Illumination Microscopy (SIM) was performed on a Zeiss Elyra PS.1 microscope (×63/1.4 NA objective) using the Zeiss ZEN 2 software with SR-SIM module.

### Immuno-electron microscopy

Immuno-electron microscopy was performed by cryosectioning and immunolabeling^40^. In brief, HeLa cells transiently expressing GFP-TECPR1 were fixed in two steps, first with 8% paraformaldehyde (PFA) and 0.4% glutaraldehyde (GA) in PHEM buffer for 5 min at room temperature and second with 4% PFA/0.2% GA in PHEM buffer for 30 min at room temperature. Cells were embedded in 2% gelatin, infused with 2.3 M sucrose at 4 °C, mounted onto pins and plunge-frozen in liquid nitrogen. Ultrathin cryosections were cut using a Leica EM FC6 cryo-ultramicrotome. Sections were immunolabeled with anti-GFP (1:50) and protein A conjugated to 10 nm gold. Samples were viewed on a Philips CM120 BioTwin Transmission Electron Microscope.

### Correlative light electron microscopy

For CLEM experiments, HeLa wt or TECPR1^KO^ cells were grown on carbon-coated sapphire discs and transfected with fluorescently tagged proteins. Cells were cryofixed using a HPM-010 High Pressure Freezing Machine (ABRA Fluid) and frozen samples were embedded in Lowicryl HM20 using a Leica EM AFS2. Freeze substitution was performed at −90 °C for 11 h with 0.1% uranyl acetate in acetone. The temperature was then raised to −45 °C at 5 °C/h and stayed at −45 °C for 5 h. Samples were washed three times with acetone and infiltrated with increasing concentrations (10%, 25%, 50%, 75%, 4 h each) of Lowicryl HM20 while the temperature was further increased to −25 °C. 100% Lowicryl HM20 was exchanged three times (10 h each) and UV polymerized at −25 °C for 48 h, followed by raising the temperature to 20 °C at 5 °C/h. Sections of 70 or 300 nm were cut on a Leica EM UC7 ultramicrotome and picked up on carbon-coated mesh grids. TetraSpeck Microspheres (100 nm, Invitrogen) were used as fiducial markers and adhered to the sections. Fluorescent microscopy imaging was performed using a widefield Olympus IX81 microscope equipped with an Olympus PlanApo ×100/1.40NA oil immersion objective^41^. Images were collected with GFP- and Cy3-specific settings. Grids carrying 70 nm sections were post-stained with 2% uranyl acetate and lead citrate and imaged on a Philips CM120 BioTwin Transmission Electron Microscope. Grids with 300 nm sections were incubated with protein A conjugated to 10 nm gold on both sides of the grid as tomographic fiducial markers, followed by post-staining with 2% uranyl acetate and lead citrate. Tomograms were collected on a TECNAI F30 Transmission Electron Microscope (FEI company) as dual-axis tilt series over a −60° to 60° tilt range with 1° increment and a magnification of ×9400 or ×15,500. A montage of the whole cell was created with lower magnification to correlate the electron micrograph with the fluorescent images using fiducial markers.

### Image analysis

Confocal and superresolution images were analyzed using Fiji. The number of GFP-hATG8 puncta was counted from Z-projections. Intensities of Alexa Fluor 488 on GUVs were normalized to the intensity of InSpeck fluorescent beads. Colocalization analysis was performed by calculating the Pearson’s correlation coefficient of single cells using a custom-made script based on the Fiji plugin Coloc2. EM tomograms were reconstructed using the IMOD software package. EM and fluorescent images were correlated using the ec-CLEM plugin of the software icy.

### Proteinase K protection assay

GFP-LC3C transfected cells were harvested and resuspended in homogenization buffer (HB: 20 mM Tris-HCl pH 8.0, 140 mM NaCl, 250 mM sucrose, 1 mM EDTA) followed by mechanical lysis by passing the suspension 15 times through a 27 gauge syringe. The nuclear fraction was separated by centrifuging at 7700 × *g* for 5 min. The supernatant was subjected to ultracentrifugation at 100,000 × *g* for 30 min. The corresponding pellet was resuspended in HB buffer and incubated with Proteinase K at a final concentration of 100 µg/ml in the presence or absence of Triton X-100 (0.5%) for the indicated times. The reaction was stopped by addition of 1 mM phenylmehtylsulfonyl fluoride and analyzed by immunoblotting.

### Co-immunoprecipitation assays

HeLa cells expressing either GFP-TECPR1 or GFP were transfected with plasmids containing HA-tagged variants of human ATG8 proteins. Cells were harvested 24 h post transfection and resuspended in lysis buffer (LB: 10 mM Hepes pH = 7.5, 0.22 M Mannitol, 0.07 M Sucrose, PMSF 100 nM). Cells were lysed by passing the suspension 30 times trough a 27 gauge syringe. Cellular debris was removed by centrifugation at 2000 × *g* for 10 min. Supernatant was incubated with GFP Trap magnetic beads (CHROMOTEK, gtma-20) overnight at 4 °C. Beads were washed using washing buffer (WB: 10 mM Tris-HCl pH = 7.5, 150 mM NaCl, 0.5 mM EDTA) and resuspended in 2× SDS buffer. Samples were analyzed by immunoblotting using antibodies as indicated.

### Statistical analysis and reproduciblitiy

Data are expressed as mean ± standard deviation (SD) of at least three independent experiments or at least 20 cells. Statistical parameters including statistical significance and *n* value are reported in the Figs. or Fig. legends. Box plots were generated using the OriginPro 9.1G software and bottom and top of the box represent the first (25%) and third (75%) quartiles, respectively. For statistical comparison of two groups of samples, the two-tailed unpaired *t*-test was used. *P*-values of < 0.05 were considered statistically significant and are indicated as following: *P < 0.05; **P < 0.01; ***P < 0.001. The exact *P*-values are given in the Source Data file.

All experiments shown are derived from two to three independent experiments (Figs. 1a, d, 2c, d, 3b, c, 4a–d, 5c, 6b, c, 7b, Supplementary Figs. 1c, d, 3a–f, 6d–f, 7g, *k* = three independent experiments; Figs. 2a, b, 6a, Supplementary Figs. 1e–g, 2a, c, 4a–c, e–g, 5a–f, 6a, *b* = two independent experiments, Supplementary Figs. 6c, 7b, *d* = one experiment).

### Reporting summary

Further information on research design is available in the Nature Research Reporting Summary linked to this article.

## Supplementary information

Supplementary Information

Peer Review File

Movie 1

Movie 2

Movie 3

Movie 4

Movie 5

Movie 6

Movie 7

Movie 8

Movie 9

Movie 10

Supplementary movie legends

Reporting summary

## Data Availability

All data generated or analyzed during this study are included in this published article (and its Supplementary information files). Data sharing is not applicable to this article as no datasets were generated or analyzed during the current study. The source data underlaying Figs. 1a–c, 2c, d, 3a, 5b, d, e, 6d, 7d and Supplementary Figs. 1a, b, 2b, c, 4d, 5c, 6c, e, h, 7b, d, g, k are provided as a Source Data file. Source data are provided with this paper.

## Source data

Source Data
