# Supplementary material for: TECPR1 promotes aggrephagy by direct recruitment of LC3C autophagosomes to lysosomes
Source: Nat Commun. 2020 Jun 12;11:2993. doi: 10.1038/s41467-020-16689-5 (PMC7293217; doi:10.1038/s41467-020-16689-5)
Supplement: Supplementary file 1 — Supplementary Information [file 41467_2020_16689_MOESM1_ESM.pdf]

Supplementary Information

TECPR1 promotes aggrephagy by direct recruitment of LC3C autophagosomes to lysosomes

Lisa Wenzel[1,2], Stéphane Blanchard[1], Sowmya Rama[1], Viola Beier[2], Anna Kaufmann[2] & Thomas Wollert[1*]

[1] Membrane Biochemistry and Transport, UMR3691 CNRS, Institute Pasteur, 28 rue du Dr Roux, 75015 Paris, France

[2] Molecular Membrane and Organelle Biology, Max Planck Institute of Biochemistry, Am Klopferspitz 18, 82152 Martinsried, Germany

Correspondence: thomas.wollert@pasteur.fr

Supplementary Information contains 7 Supplementary Figures including legends, Supplementary Movie legends and Supplementary Table 1.

Supplemental Figures

Supplementary Figure 1: Subcellular localization of TECPR1.

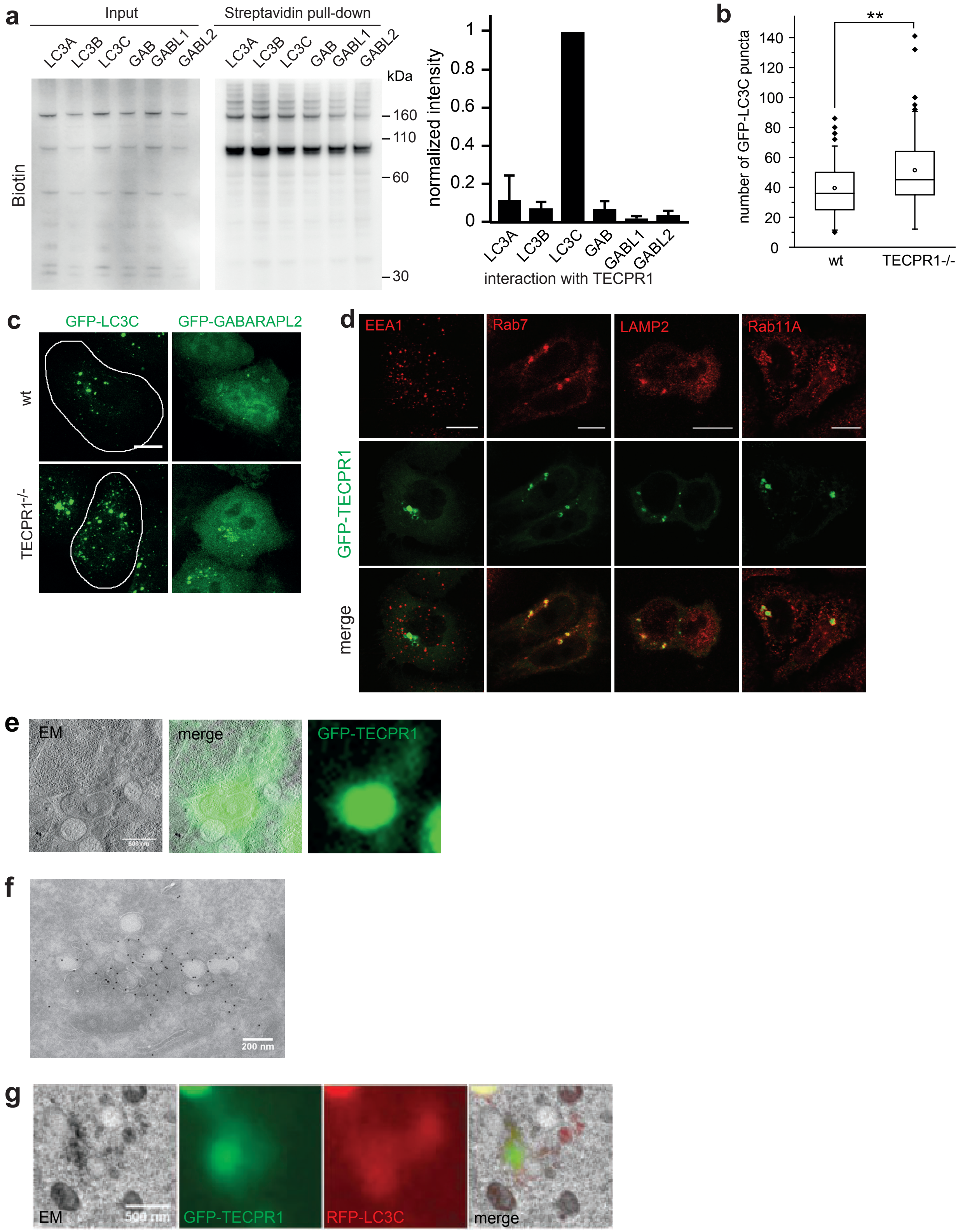

(a) Biotin and Streptavidin-HRP immunoblots of samples from BioID assay and quantification of immunoblot signals from HA-tagged hATG8s as shown in Fig. 1a. Intensities in pull-down samples were corrected for the corresponding input sample intensities and normalized to that of LC3C which was set to one (n=3). Data are represented as mean ± SD. **(b)** The number of GFP-LC3C puncta was counted in non-starved wildtype (wt) or TECPR1$^{-/-}$ cells (n > 50 cells pooled from three independent experiments). Box plots represent the first (25%) and third (75%) quartiles, respectively. The centre line represents the median, whiskers the standard deviation and minima and maxima are available from the Source Data file, n = 28 cells. P values were calculated using a two-tailed Student's t-test (**P < 0.01, ***P < 0.001). A significant accumulation of LC3C puncta was observed in cells lacking TECPR1. **(c)** Confocal images of starved HeLa and TECPR1cells$^{-/-}$ cells expressing GFP-LC3C or GFP-GABARAP as indicated. Images correspond to quantification shown in Figure 1c. Scale bar = 10 µm. **(d)** Confocal images of starved HeLa cells expressing GFP-TECPR1 that were immunostained for EEA1, Rab7, LAMP2, or Rab11A as indicated. TECPR1 strongly colocalized with Rab7 and LAMP2. A limited colocalization of TECPR1 with Rab11A was observed. TECPR1 was not detected at EEA1 positive compartments. Scale bars, 10 µm. **(e)** Correlative light electron micrographs of starved HeLa cells expressing GFP-TECPR1. Tomograms of 300 nm sections were acquired and one representative slice of the tomogram and the correlated fluorescent image are depicted. Scale bar, 500 nm. **(f)** Immuno-EM of starved HeLa cells expressing GFP-TECPR1. Cryo-sections were stained with anti-GFP (10 nm gold). Scale bar, 200 nm. **(g)** Correlative light electron micrographs of starved HeLa cells expressing GFP-TECPR1 and RFP-LC3C. Tomograms of 300 nm sections were acquired and one representative slice of the tomogram and the correlated fluorescent image are depicted. Scale bar, 500 nm. See also Figure 1. Source data are provided as a Source Data file.

Supplementary Figure 2: Colocalization of TR1 with LC3C.

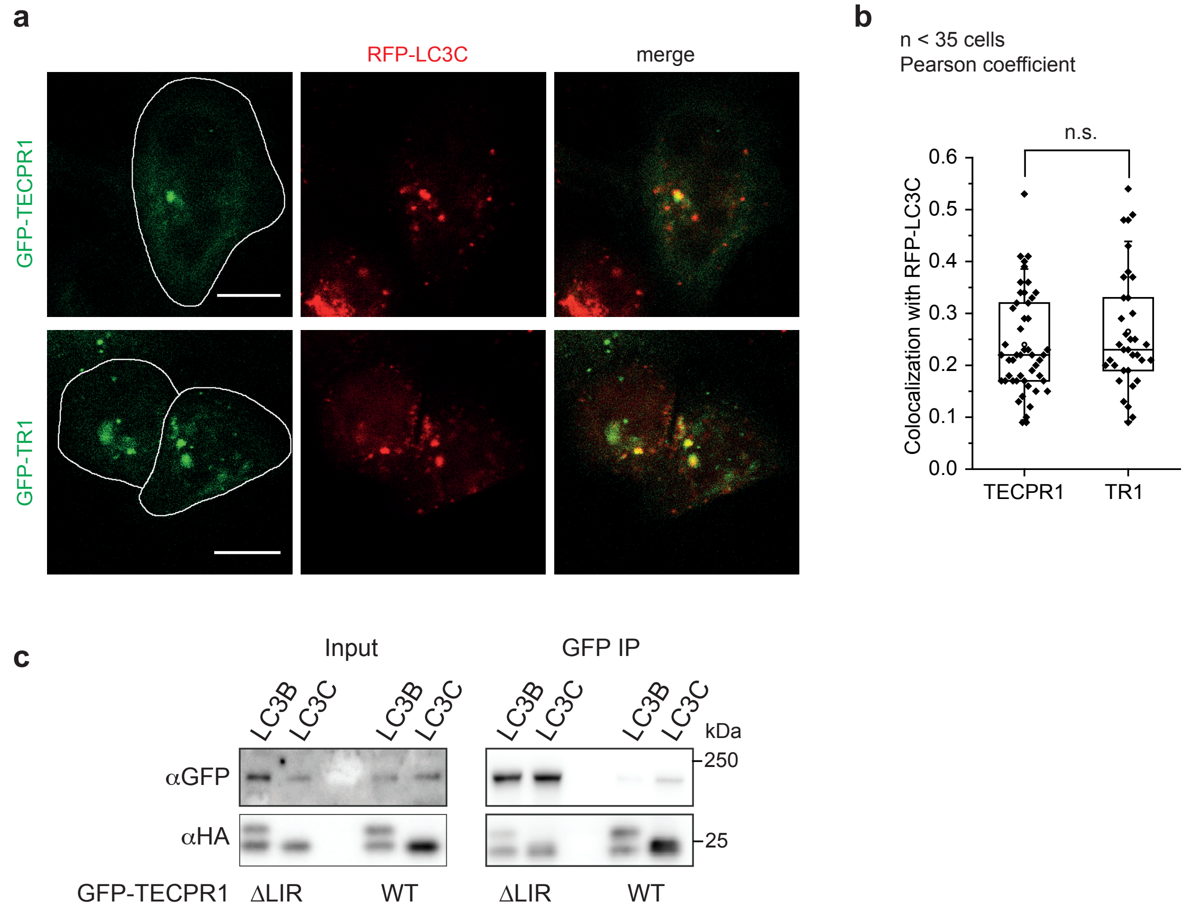

(a) Representative confocal images of HeLa cells expressing RFP-LC3C and either GFP-TECPR1 or GFP-TR1. Both, TECPR1 and TR1 colocalize with LC3C. Scale bars, 10 µm. **(b)** Quantification of the colocalization between TECPR1 or TR1 with LC3C using the Pearson coefficient. Box plots represent the first (25%) and third (75%) quartiles, respectively. The centre line represents the median, whiskers the standard deviation and minima and maxima are available from the Source Data file, n = 90 cells pooled from three independent experiments. P values were calculated using a two-tailed Student's t-test. No statistically significant difference in the colocalization of TR1 or TECPR1 is observed. **(c)** Coimmunoprecipitation of HA-tagged LC3B or LC3C as indicated from lysates of cells expressing GFP-TECPR1 (WT) or GFP-TECPR1$^{\Delta LIR}$ (TECPR1$^{W175A/I178A}$, ΔLIR) using the GFP-Trap reagent. Western blots of lysates and co-IPs are shown. See also Figure 2. Source data are provided as a Source Data file.

Supplementary Figure 3: Colocalization of TECPR1 with LC3C.

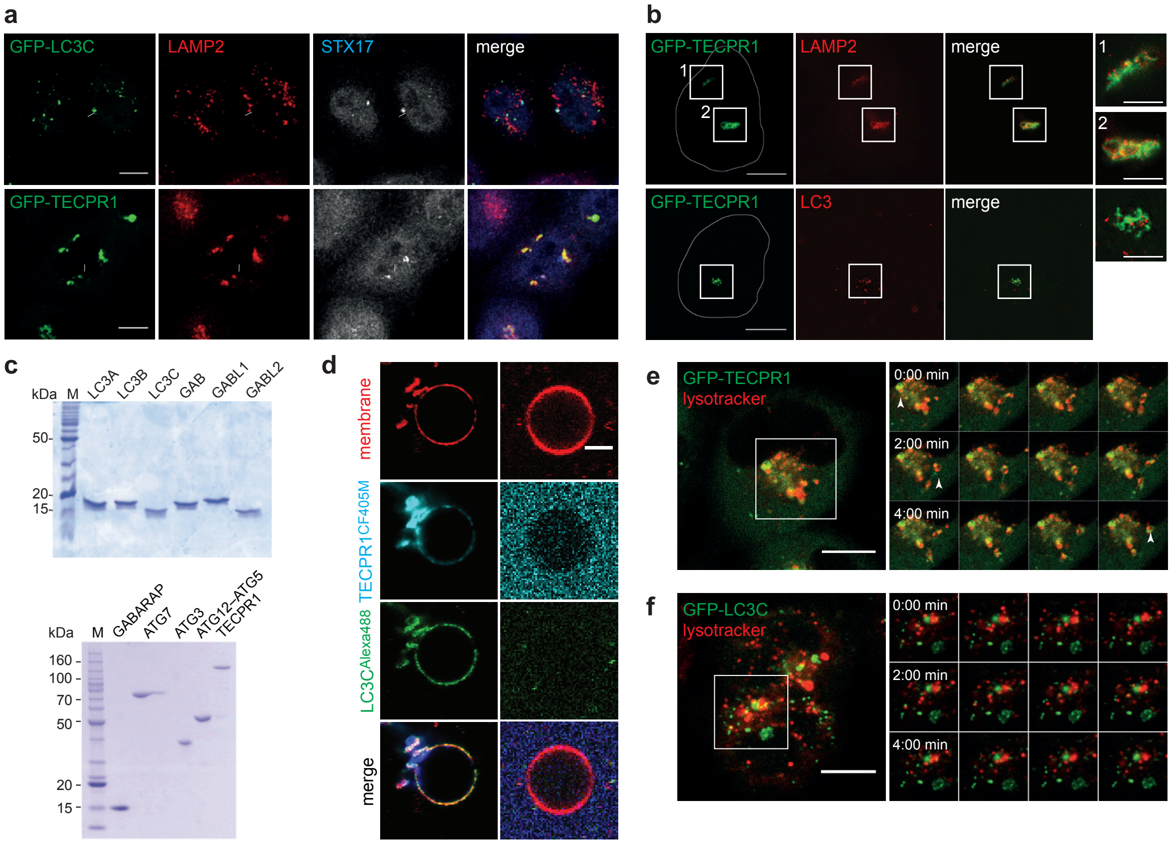

(a) Representative confocal images of HeLa cells expressing either GFP-LC3C or GFP-TECPR1 and immunostained using LAMP2 or Syntaxin17 (STX17) selective antibodies. LC3C preferentially colocalizes with the late autophagosomal marker STX17 whereas TECPR1 colocalizes with the lysosomal marker LAMP2. Images correspond to the quantitative analysis shown in Figure 3A. Scale bars, 10 µm. **(b)** Structured-illumination microscopy of starved HeLa cells expressing GFP-TECPR1 and immunostained for LAMP2 or LC3 as indicated. TECPR1 colocalizes with LAMP2, while LC3 puncta are in the immediate vicinity of TECPR1 compartments. Scale bars, 10 µm, insets, 2.5 µm. **(c)** SDS-PAGE of purified recombinant proteins including the six hATG8s (upper gel) as well as the Ub-like conjugation system including TECPR1 (lower gel). These proteins have been used for in vitro reconstitutions. GAB = GABARAP. **(d)** GUVs containing PtdIns(4)P and Lissamine-rhodamine-PE (red) were pre-incubated with CF405M-labeled TECPR1 and mixed with SUVs to which Alexa488-labeled LC3C was conjugated. Representative confocal images of GUVs that were positive (left panel) or negative (right panel) for TECPR1 are depicted. LC3C vesicles only accumulated at GUVs to which TECPR1 was bound. Scale bar, 3 µm. **(e,f)** Time-lapse video microscopy of HeLa cells treated with lysotracker deep red and expressing GFP-TECPR1 (**e**) or GFP-LC3C (**f**). Confocal images were captured 30 min after lysotracker treatment with intervals of 30 seconds. Insets show the marked region of confocal frames at indicated time points. Arrowheads indicate ring-like TECPR1 structures containing lysotracker. Scale bars, 10 µm. See also Figure 3 and Supplementary Movies 1, 2, 3 and 4.

Supplementary Figure 4: Binding of TECPR1 to PtdInsPs.

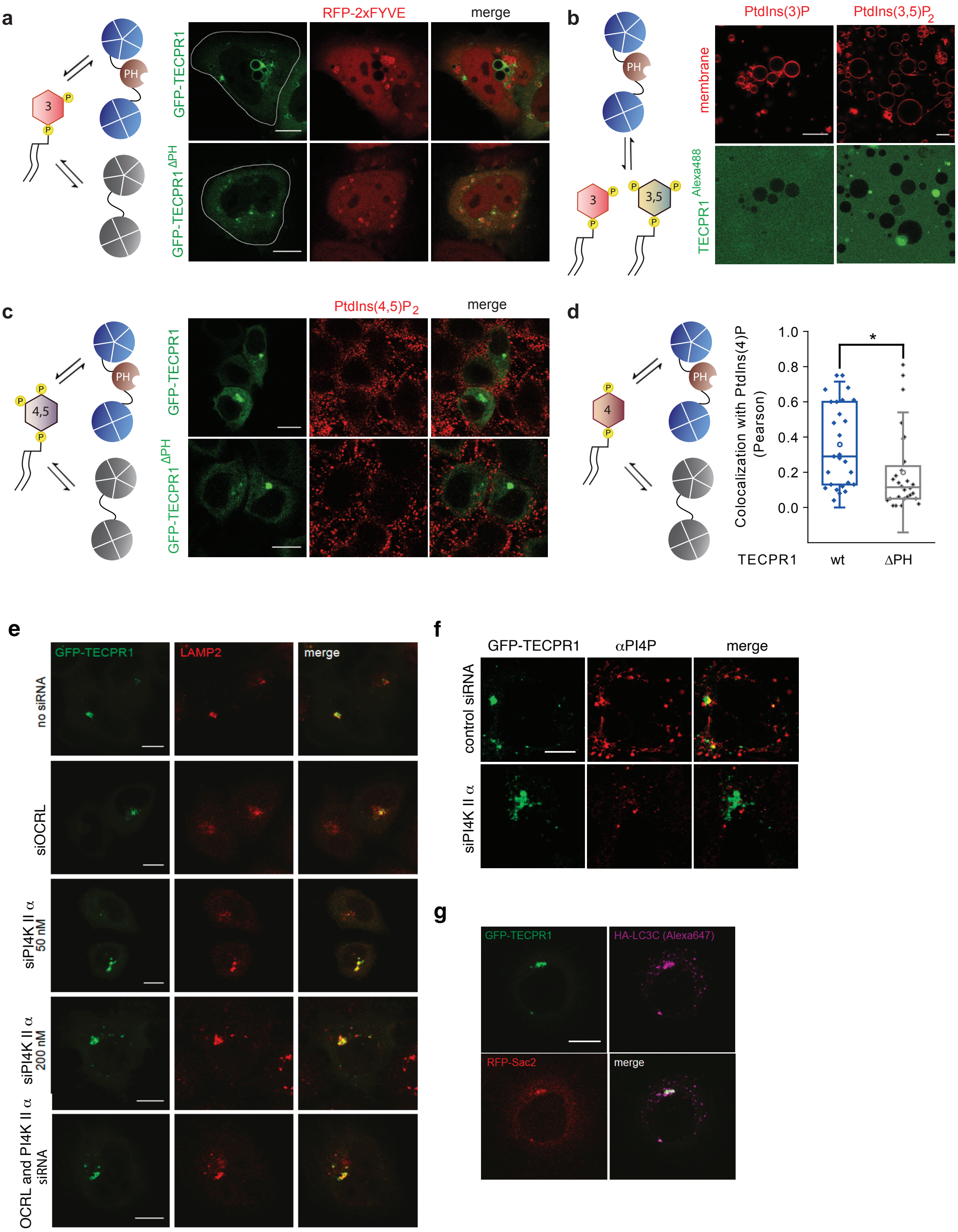

(a) Representative confocal images of TECPR1$^{-/-}$ cells coexpressing either GFP-TECPR1 or GFP-TECPR1$^{\Delta PH}$ with the PtdIns(3)P-sensor RFP-2xFYVE. Scale bars, 10 µm. **(b)** Confocal images of Alexa488-labeled TECPR1 (green) incubated with GUVs containing Lissamine-rhodamine-PE and either PtdIns(3)P or PtdIns(3,5)P_2 (red). TECPR1 is not recruited to GUVs. Scale bar = 20 µm. **(c)** Colocalization of GFP-TECPR1 or GFP-TECPR1$^{\Delta PH}$ with immunostained PtdIns(4,5)P_2 in TECPR1$^{-/-}$ cells. Scale bars, 10 µm. Neither TECPR1 nor TECPR1$^{\Delta PH}$ colocalize with PtdIns(3)P or PtdIns(4,5)P_2. Scale bar, 20 µm. **(d)** Quantitative analysis of the colocalization of GFP-TECPR1 or GFP-TECPR1$^{\Delta PH}$ with immunostained PtdIns(4)P in TECPR1$^{-/-}$ cells as shown in Figure 4c. Box plots represent the first (25%) and third (75%) quartiles, respectively. The centre line represents the median, whiskers the standard deviation and minima and maxima are available from the Source Data file, n = 45 cells pooled from three independent experiments. P values were calculated using a two-tailed Student's t-test (*$P < 0.05$). **(e)** Confocal images of HeLa cells expressing GFP-TECPR1 and immunostained for LAMP2, treated with siRNAs against PI4KIIα (Phosphatidylinositol(4)-kinase 2 alpha) and OCRL (Phosphatidylinositol(5)-phosphatase) or both. Scale bar = 10 µm. **(f)** Confocal images of HeLa cells expressing GFP-TECPR1, immunostained for PtdIns(4)P and treated with siRNA against PI4KIIα or control siRNA as indicated. **(g)** Confocal images of HeLa cells coexpressing GFP-TECPR1, the RFP-tagged PtdIns(4) phosphatase Sac2 and HA-LC3C. Immunostaining of LC3C was done using anti HA-antibody and Alexa647-labeled secondary antibody. Scale bar = 10 µm. See also Figure 4. Source data are provided as a Source Data file.

Supplementary Figure 5: TECPR1$^{\Delta PH\text{-}2xFYVE}$ selectively recruits LC3C to MVB-like structures.

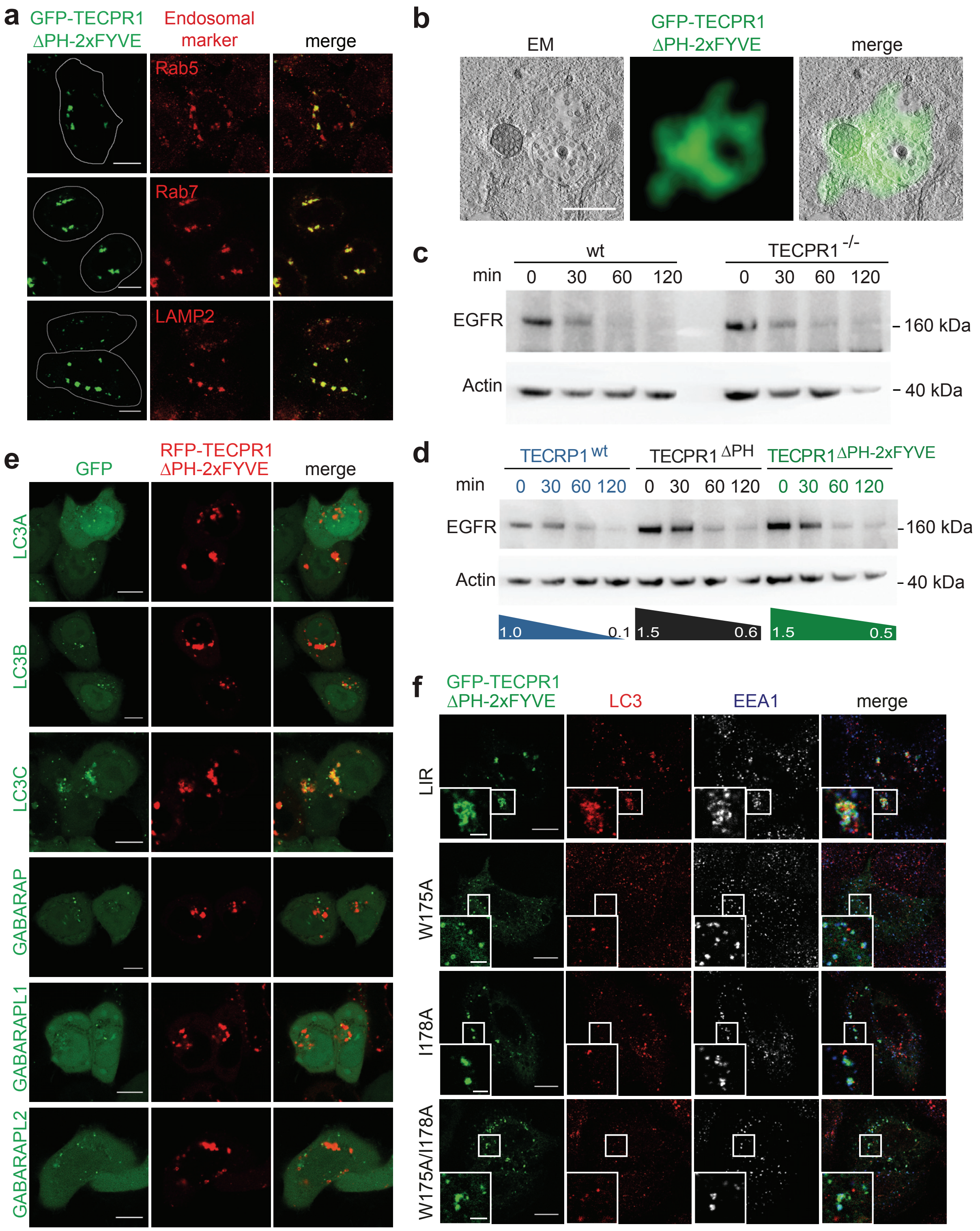

(a) Confocal images of TECPR1$^{-/-}$ cells expressing GFP-TECPR1$^{\Delta PH\text{-}2xFYVE}$ and immunostained for Rab5, Rab7 or LAMP2 as indicated. GFP-TECPR1$^{\Delta PH\text{-}2xFYVE}$ strongly colocalized with early and late endosomal markers. Scale bars, 10 µm. **(b)** Correlative light electron tomograph of GFP-TECPR1$^{\Delta PH\text{-}2xFYVE}$ expressing TECPR1$^{-/-}$ cells. Tomograms of 300 nm sections were acquired and one representative slice of the tomogram is depicted. TECPR1$^{\Delta PH\text{-}2xFYVE}$ is found on structures reminiscent of multivesicular bodies (MVBs). Scale bar, 500 nm. **(c, d)** Endosomal growth factor receptor (EGFR) degradation assay of lysates from wildtype (wt) and TECPR1$^{-/-}$ cells **(c)** as well as TECPR1$^{-/-}$ cells expressing TECPR1^{wt}, TECPR1$^{\Delta PH}$, or TECPR1$^{\Delta PH\text{-}2xFYVE}$ **(d)**. After stimulation with EGF for the indicated times, cell lysates were subjected to immunoblotting against EGFR and actin. Neither deletion of TECPR1 (**c**) nor expression of TECPR1 variants **(d)** impaired degradation of EGFR. **(d)** Band intensities were depicted as chats and numbers represent quantified EGFR intensities that were normalized to actin intensities (loading control). **(e)** Representative confocal images of TECPR1$^{-/-}$ cells coexpressing RFP-TECPR1$^{\Delta PH\text{-}2xFYVE}$ and GFP-tagged hATG8s as indicated. Only LC3C colocalizes with TECPR1$^{\Delta PH\text{-}2xFYVE}$. The corresponding quantification of colocalizations of TECPR1$^{\Delta PH\text{-}2xFYVE}$ and the indicated hATG8 is shown in Figure 5D. Scale bars, 10 µm. **(f)** Confocal images of TECPR1$^{-/-}$ cells expressing GFP-TECPR1$^{\Delta PH\text{-}2xFYVE}$ or its corresponding single LIR mutants (W175A or I178A) or double LIR mutant (W175AI178A). The colocalization of TECPR1-variants with endogenous LC3C and EEA1 was assessed by immunostaining with an LC3 antibody and an EEA1 antibody. The corresponding quantification is shown in Figure 5e. Scale bars, 10 µm, insets 2.5 µm. See also Figure 5 and Supplementary Movie 5. Source data are provided as a Source Data file.

Supplementary Figure 6: Colocalization of LC3C puncta with selective cargo markers and clearance of protein aggregates in ATG8 depleted cells.

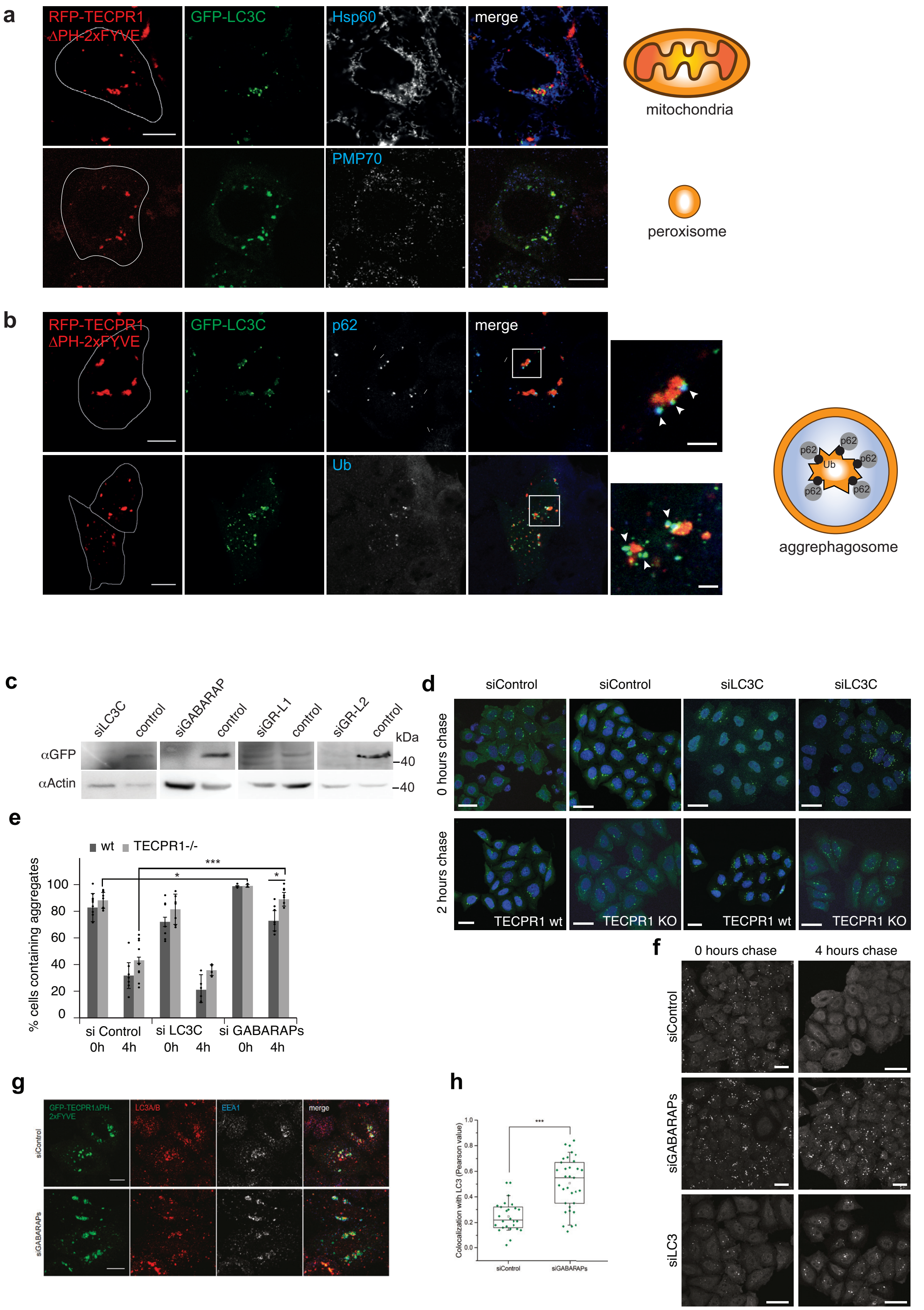

(a, b) Confocal images of TECPR1$^{-/-}$ cells coexpressing RFP-TECPR1$^{\Delta PH-2xFYVE}$ and GFP-LC3C and immunostained for Hsp60 or PMP70 (**a**), as well as p62 or Ub (**b**). (**a**) Hsp60 (mitochondrial marker) and PMP70 (peroxisomal marker) did not colocalize with LC3C or TECPR1$^{\Delta PH-2xFYVE}$ puncta. (**b**) LC3C structures colocalize with p62 and Ubiquitin (Ub) at TECPR1$^{\Delta PH-2xFYVE}$ structures. Insets show enlarged regions with arrowheads pointing to GFP-LC3C puncta that colocalized with p62 or Ub. Scale bars, 10 µm, inset, 2.5 µm. (**c**) Western blots of HeLa cell lysates expressing GFP-tagged ATG8 proteins and treated with corresponding siRNAs as indicated. Immunostaining using anti GFP-antibodies demonstrated the efficiency of ATG8-depletion. Immunoblots of actin serve as loading control. **(d)** TECPR1^{WT} and TECPR1^{KO} cells were treated with siRNAs as indicated for 24 ours followed by treatment with puromycin for 2 hours. The clearance of protein aggregates was followed by a chasing phase for 2 hours during which proteasomal degradation of proteins was blocked by addition of MG132. Autophagic degradation of protein aggregates is impaired in TECPR1^{KO} cells, upon knockdown of LC3C, and in LC3C depleted TECPR1^{KO} cells. **(e)** Quantification of Ub puncta in TECPR1^{WT} and TECPR1^{KO} cells, treated with siRNA as indicated. Protein aggregation was induced by puromycin treatment for 2 hours followed by a recovery period of 4 hours. Data are shown as mean ± SD of three independent experiments. P values were calculated using a two-tailed Student's t-test (*P < 0.05, ***P < 0.001). **(f)** HeLa cells were treated with siRNAs as indicated for 72 h and incubated with puromycin for 4 hours followed by a recovery phase of 4 hours. Autophagic degradation of protein aggregates is strongly impaired in siGABARAP cells. **(g)** Confocal images of HeLa cells were treated with siRNA against GABARAPs or control siRNA and transfected with GFP-TECPR1$^{\Delta PH-2xFYVE}$. Fixed cells were immunostained with a nonselective LC3 antibody and an EEA1 antibody. A significant accumulation of LC3 puncta at EEA1-positive GFP-TECPR1$^{\Delta PH-2xFYVE}$ structures was observed if GABARAPs were knocked down. Scale bar = 10 µm. **(h)** Quantification of the colocalization of LC3 positive structures with TECPR1 and EEA1 from images as shown in **(g)** using the Pearson coefficient. Box plots represent the first (25%) and third (75%) quartiles, respectively. The centre line represents the median, whiskers the standard deviation and minima and maxima are available from the Source Data file, n = 26 cells pooled from three independent experiments. P values were calculated using a two-tailed Student's t-test (***P < 0.001). See also Figure 6. Source data are provided as a Source Data file.

Supplementary Figure 7: TECPR1 promotes aggrephagy in neural stem cells.

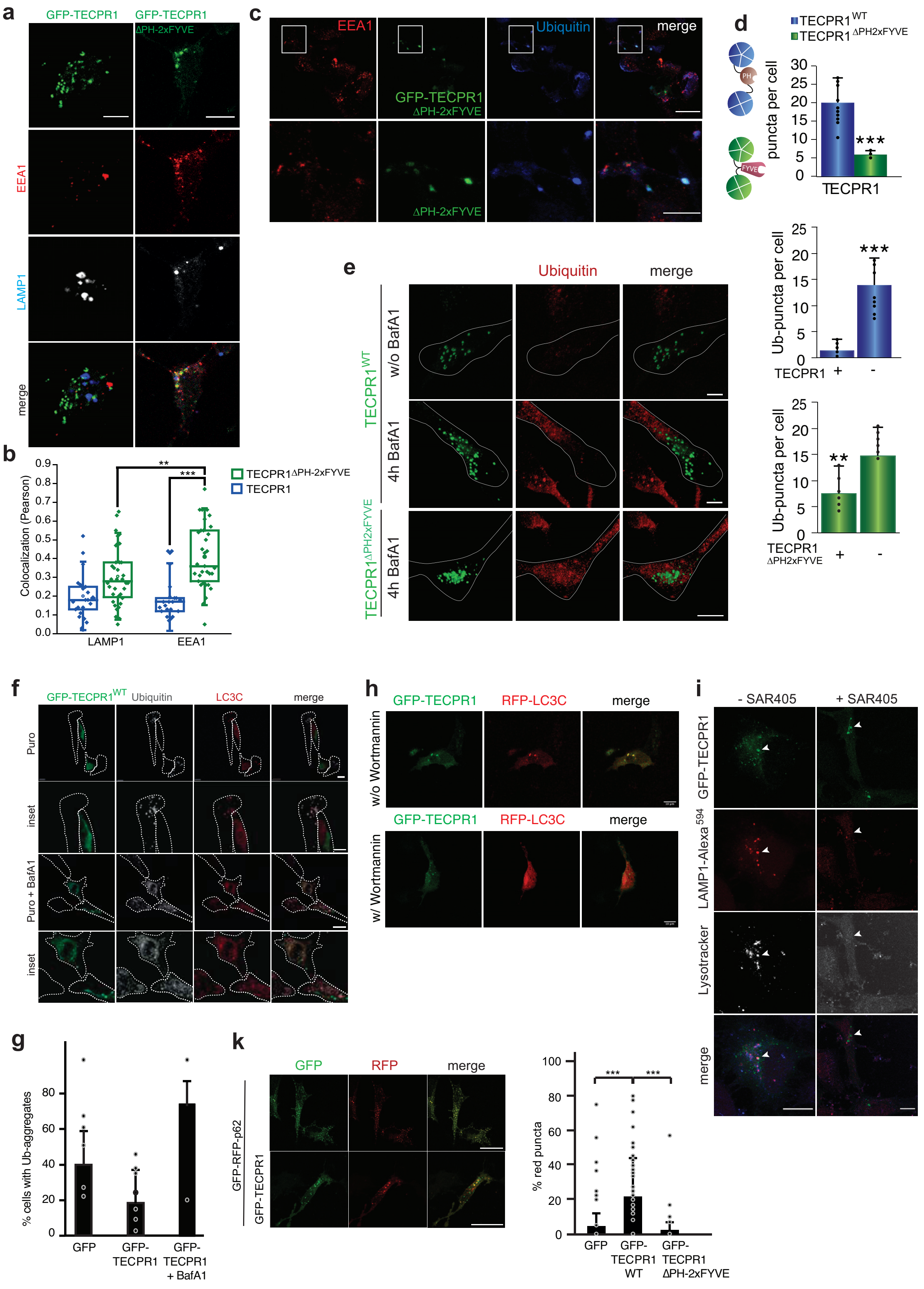

(a) Confocal images of neural stem cells (NSCs) expressing GFP-TECPR1 or GFP-TECPR1$^{\Delta PH\text{-}2xFYVE}$ and immunostained against EEA1 and LAMP1. TECPR1 colocalizes with LAMP1 whereas TECPR1$^{\Delta PH\text{-}2xFYVE}$ was detected on EEA1 positive structures. Scale bars, 10 µm. (**b)** Quantification of the colocalization of GFP-TECPR1 or GFP-TECPR1$^{\Delta PH\text{-}2xFYVE}$ with LAMP1 or EEA1 in NSCs as shown in (**a**). A significant stronger colocalization of TECPR1$^{\Delta PH\text{-}2xFYVE}$ with EEA1 compared to LAMP1 was observed. Moreover, TECPR1$^{\Delta PH\text{-}2xFYVE}$ colocalized significantly stronger with EEA1 compared to TECPR1. Box plots represent the first (25%) and third (75%) quartiles, respectively. The centre line represents the median, whiskers the standard deviation and minima and maxima are available from the Source Data file, n = 20 cells pooled from three independent experiments. P values were calculated using a two-tailed Student's t-test (**P < 0.01, ***P < 0.001). **(c)** Confocal images of neural stem cells expressing GFP-TECPR1$^{\Delta PH\text{-}2xFYVE}$ that were immunostained for EEA1 and Ubiquitin. The lower panel shows a magnification of the area that is indicated in the upper panel. See also Figure 7a. **(d)** Quantification of TECPR1 and Ub puncta of images as shown in (**d** and Figure **7a**) with data being represented as mean ± SD. Data are based on quantification of 15 cells pooled from three independent experiments. P values were calculated using a two-tailed Student's t-test (**P < 0.01, ***P < 0.001). **(e)** Confocal images of NSCs expressing GFP-TECPR1 or GFP-TECPR1$^{\Delta PH\text{-}2xFYVE}$ that were immunostained for Ubiquitin. Cells were treated with Bafilomycin A1 (BafA1) as indicated. **(f)** Confocal images of puromycin treated NSCs coexpressing GFP-TECPR1 and RFP-LC3C that have been immunostained for Ubiquitin. BafA1 treated or non-treated cells are shown as indicated. BafA1 inhibits TECPR1-dependent reduction of LC3C-positive Ubiquitin puncta. **(g)** Quantification of the percentage of NSCs with Ub-aggregates. NPCs were transfected with GFP or GFP-TECPR1 as indicated and treated with puromycin for 2 hours. Autophagy was inhibited by incubating GFP-TECPR1 transfected cells with BafA1 for 4 hours. Data are shown as mean with SD of three independent experiments based on 50 cells each. **(h)** Confocal images of NPCs coexpressing GFP-TECPR1 and RFP-LC3C were treated with the PI3-kinase inhibitor wortmannin as indicated. The formation of LC3C puncta is inhibited in wortmannin treated cells. **(i)** Confocal images of NPCs expressing GFP-TECPR1 and immunostained for LAMP1 using an anti LAMP1 primary and an Alexa594 labeled secondary antibody were treated with lysotracker deep red and the VPS34 specific inhibitor SAR405 as indicated. Arrowheads indicate the position of TECPR1 structures. Scale bars, 10 µm. (**k**) Representative confocal images of NSCs expressing GFP-RFP-p62 with or without GFP-TECPR1. The chart shows the corresponding quantitative analysis of RFP-puncta. Puncta with colocalizing red and green fluorescence indicate p62 aggregates in the cytoplasm whereas puncta with only red fluorescence are located within lysosomes. Quantification of three independent experiments are shown as mean ± standard deviation. P values were calculated using a two-tailed Student's t-test (**P < 0.01, ***P < 0.001). See also Figure 7 and Supplementary Movie 6. Source data are provided as a Source Data file. All scale bars in the figure correspond to 10 µm.

Supplemental Movie Legends:

Movie 1: TECPR1 recruits LC3C to lysosomes.
Structured illumination microscopy of HeLa cells coexpressing RFP-TECPR1 and GFP-LC3C and immunolabeled for LAMP2 (in blue). The movie shows a 3D reconstructed z-stack. Corresponds to Figure 3B.

Movie 2: LC3C vesicles are recruited to TECPR1 structures.
Time-lapse video microscopy of TECPR1$^{-/-}$ cells coexpressing RFP-TECPR1 and GFP-LC3C. 16 ± 2 % of TECPR1 structures are large (>1.0 µm) and move slowly, the remaining structures are small (usually <0.5 µm) and traffic faster. The arrowhead indicates a LC3 puncta that associates with a TECPR1 structure. Related to Figure 3C.

Movie 3: TECPR1 structures correspond to acidified organelles.
Time-lapse video microscopy of HeLa cells expressing GFP-TECPR1, which have been treated with lysotracker deep red. 25 % of lysotracker positive structures colocalize with TECPR1. Theses lysosomes appear to be larger (>1 µm) and less mobile than the smaller structures that are TECPR1 negative. The circular TECPR1 structures that surrounds a lysotracker puncta is labeled with an arrowhead. Related to Supplementary Figure 2E.

Movie 4: LC3C vesicles are recruited to acidified organelles.
Time-lapse video microscopy of HeLa cells expressing GFP-LC3C, which have been treated with lysotracker deep red. The majority of LC3C puncta is small (85%, < 0.5 µm) and mobile. A LC3C puncta that traffics towards and associates with a lysotracker positive structure (lysotracker structures are < 1 µm in size and immobile) is indicted by an arrowhead. Related to Supplementary Figure 2F.

Movie 5: TECPR1$^{\Delta PH\text{-}2xFYVE}$ reroutes LC3C vesicles to endosomes.
Structured illumination microscopy of HeLa cells coexpressing RFP-TECPR1$^{\Delta PH\text{-}2xFYVE}$ and GFP-LC3C and immunolabeled for EEA1 (in blue). The movie shows a 3D reconstructed z-stack. Corresponds to Figure 5C.

Movie 6: Overexpression of LC3B does not outcompete LC3C from protein aggregate containing autophagosomes.
3D reconstruction of deconvoluted confocal z-stacks of neural stem cells coexpressing GFP-LC3C and RFP-LC3B. Cells were immunostained using an anti-Ubiquitin antibody. Corresponds to Figure 7E.

Movie 7: LC3C puncta traffic to TECPR1 structures and fuse with them.
NSCs were transfected with GFP-TECPR1and RFP-LC3C. Z-stacks of cells were recorded over indicated times. The movie shows 3D volumes of one cell at different time points. Volumes were reconstructed from z-stacks using Fiji. The arrowhead indicates a TECPR1-positive structure that fuses with LC3C puncta.

Movie 8: LC3C puncta fuse with TECPR1 structures.
The movie shows the same cell that is shown in Movie 7 at one representative time-point. The reconstructed 3D volume rotates by 360° to visualize the association of TECPR1 and LC3C structures shortly before fusion.

Movie 9: LC3C puncta associate with TECPR1ΔPH-2xFYVE structures but did not fuse.
NSCs were transfected with GFP-TECPR1ΔPH-2xFYVE and RFP-LC3C. Z-stacks of cells were recorded over indicated times. The movie shows 3D volumes of one cell at different time points. Volumes were reconstructed from z-stacks using Fiji. The arrowhead indicates a TECPR1 PH-2xFYVE -positive structure that associates with an LC3C puncta. No fusion was observed over the recorded time period.

Movie 10: LC3C puncta are recruited to TECPR1ΔPH-2xFYVE structures.
The movie shows the same cell that is shown in Movie 8 at one representative time-point. The reconstructed 3D volume rotates by 360° to visualize the association of TECPR1ΔPH-2xFYVE and LC3C structures.

Supplementary Table 1: Genotyping results of CRISPR/Cas9 HeLa knockout cell line. Deletions are indicated in red, insertions in blue, and stop codons in bold. The resulting changes in the amino acid sequences are highlighted in yellow.

**Name**	**CRISPR guide RNA (PAM)**	**Genomic Mutations**	**Protein translation**
Gene name<br>*TECPR1*<br><br>Uniprot name<br>*Q7Z6L1*<br><br>Clone name<br>*TECPR1 KO sgRNA4 A3*	GCGTGTACAC<br>TCTCCCGAAG<br>AGG	Allele 1 (first exon…..second exon):<br>ATGCCCAACTCAGTGCTGTGG<br>CGGTGGACCTCTTCGGGAGAGT<br>GTACACGCTGTCCACAGCAGGC<br>CAGTACTGGGAAATGTGCAAGG<br>ACTCCCAGCTGGAGTTCAAGCG<br>CGTCAGCGCCACCACGCAGTGC<br>TGCTGGGGCATTGCCTGTGACA<br>ACCAGGTCTACGTGTATGTGTG<br>TGCCAGCGATGTCCCCATCCGC<br>CGCCGAGAGGAGGCCTATGAG<br>AATCAG……CGCTGGAATCCCA<br>TGGGCGGCTTCTGTGAGAAGCT<br>CCTGC**TGA**<br><br>Allele 2 (first exon):<br>ATGCCCAACTCAGTGCTGTGG<br>CGGTGGACCTCTTCACTAAAAG<br>GGCTGTTCCAGCCGTCCGTGCT<br>GGCGCCTGGGGATCCAGGCCA<br>GTTCCCGGTACCCGCCCATGCC<br>CAGCCCGGCGGCCTAGGGCGT<br>TACCT**TGA**	MPNSVLWAVDLLRCP<br>QQASTGKCARTPSWS<br>SSASAPPRSAAGALPV<br>TTRSTCMCVPAMSPS<br>AAERRPMRISAGIPWA<br>ASVRSSC-<br><br>MPNSVLWAVDLFTKRA<br>VPAVRAGAWGSRPVP<br>GTRPCPARRPRALP-
