# Supplementary material for: TECPR1 promotes aggrephagy by direct recruitment of LC3C autophagosomes to lysosomes
Source: Nat Commun. 2020 Jun 12;11:2993. doi: 10.1038/s41467-020-16689-5 (PMC7293217; doi:10.1038/s41467-020-16689-5)
Supplement: Supplementary file 13 — Supplementary movie legends [file 41467_2020_16689_MOESM13_ESM.docx]

**Supplemental Movie Legends:**

**Movie 1: TECPR1 recruits LC3C to lysosomes.**

Structured illumination microscopy of HeLa cells coexpressing RFP-TECPR1 and GFP-LC3C and immunolabeled for LAMP2 (in blue). The movie shows a 3D reconstructed z-stack. Corresponds to Figure 3B.

**Movie 2: LC3C vesicles are recruited to TECPR1 structures.**

Time-lapse video microscopy of TECPR1^-/-^ cells coexpressing RFP-TECPR1 and GFP-LC3C. 16 ± 2 % of TECPR1 structures are large (>1.0 µm) and move slowly, the remaining structures are small (usually <0.5 µm) and traffic faster. The arrowhead indicates a LC3 puncta that associates with a TECPR1 structure. Related to Figure 3c.

**Movie 3: TECPR1 structures correspond to acidified organelles.**

Time-lapse video microscopy of HeLa cells expressing GFP-TECPR1, which have been treated with lysotracker deep red. 25 % of lysotracker positive structures colocalize with TECPR1. Theses lysosomes appear to be larger (>1 µm) and less mobile than the smaller structures that are TECPR1 negative. The circular TECPR1 structures that surrounds a lysotracker puncta is labeled with an arrowhead. Related to Supplementary Figure 2e.

**Movie 4: LC3C vesicles are recruited to acidified organelles.**

Time-lapse video microscopy of HeLa cells expressing GFP-LC3C, which have been treated with lysotracker deep red. The majority of LC3C puncta is small (85%, < 0.5 µm) and mobile. A LC3C puncta that traffics towards and associates with a lysotracker positive structure (lysotracker structures are < 1 µm in size and immobile) is indicted by an arrowhead.

Related to Supplementary Figure 2f.

**Movie 5: TECPR1^∆PH-2xFYVE^ reroutes LC3C vesicles to endosomes.**

Structured illumination microscopy of HeLa cells coexpressing RFP-TECPR1^∆PH-2xFYVE^ and GFP-LC3C and immunolabeled for EEA1 (in blue). The movie shows a 3D reconstructed z-stack. Corresponds to Figure 5c.

**Movie 6: Overexpression of LC3B does not outcompete LC3C from protein aggregate containing autophagosomes.**

3D reconstruction of deconvoluted confocal z-stacks of neural stem cells coexpressing GFP-LC3C and RFP-LC3B. Cells were immunostained using an anti-Ubiquitin antibody. Corresponds to Figure 7e.

**Movie 7: LC3C puncta traffic to TECPR1 structures and fuse with them.**

NSCs were transfected with GFP-TECPR1and RFP-LC3C. Z-stacks of cells were recorded over indicated times. The movie shows 3D volumes of one cell at different time points. Volumes were reconstructed from z-stacks using Fiji. The arrowhead indicates a TECPR1-positive structure that fuses with LC3C puncta.

**Movie 8: LC3C puncta fuse with TECPR1 structures.**

The movie shows the same cell that is shown in Movie 7 at one representative time-point. The reconstructed 3D volume rotates by 360° to visualize the association of TECPR1 and LC3C structures shortly before fusion.

**Movie 9: LC3C puncta associate with TECPR1^∆PH-2xFYVE^ structures but did not fuse.**

NSCs were transfected with GFP-TECPR1^∆PH-2xFYVE^ and RFP-LC3C. Z-stacks of cells were recorded over indicated times. The movie shows 3D volumes of one cell at different time points. Volumes were reconstructed from z-stacks using Fiji. The arrowhead indicates a TECPR1 PH-2xFYVE -positive structure that associates with an LC3C puncta. No fusion was observed over the recorded time period.

**Movie 10: LC3C puncta are recruited to TECPR1^∆PH-2xFYVE^ structures.**

The movie shows the same cell that is shown in Movie 8 at one representative time-point. The reconstructed 3D volume rotates by 360° to visualize the association of TECPR1^∆PH-2xFYVE^ and LC3C structures.
