# Supplementary material for: TECPR1 promotes aggrephagy by direct recruitment of LC3C autophagosomes to lysosomes
Source: Nat Commun. 2020 Jun 12;11:2993. doi: 10.1038/s41467-020-16689-5 (PMC7293217; doi:10.1038/s41467-020-16689-5)
Supplement: Supplementary file 14 — Reporting summary [file 41467_2020_16689_MOESM14_ESM.pdf]

nature research

Corresponding author(s): Thomas Wollert

Last updated by author(s): 2020/05/04

Reporting Summary

Nature Research wishes to improve the reproducibility of the work that we publish. This form provides structure for consistency and transparency in reporting. For further information on Nature Research policies, see Authors & Referees and the Editorial Policy Checklist.

Statistics

For all statistical analyses, confirm that the following items are present in the figure legend, table legend, main text, or Methods section.

n/a	Confirmed	
☐	☒	The exact sample size (*n*) for each experimental group/condition, given as a discrete number and unit of measurement
☐	☒	A statement on whether measurements were taken from distinct samples or whether the same sample was measured repeatedly
☐	☒	The statistical test(s) used AND whether they are one- or two-sided<br>*Only common tests should be described solely by name; describe more complex techniques in the Methods section.*
☒	☐	A description of all covariates tested
☒	☐	A description of any assumptions or corrections, such as tests of normality and adjustment for multiple comparisons
☐	☒	A full description of the statistical parameters including central tendency (e.g. means) or other basic estimates (e.g. regression coefficient) AND variation (e.g. standard deviation) or associated estimates of uncertainty (e.g. confidence intervals)
☐	☒	For null hypothesis testing, the test statistic (e.g. *F*, *t*, *r*) with confidence intervals, effect sizes, degrees of freedom and *P* value noted<br>*Give P values as exact values whenever suitable.*
☒	☐	For Bayesian analysis, information on the choice of priors and Markov chain Monte Carlo settings
☒	☐	For hierarchical and complex designs, identification of the appropriate level for tests and full reporting of outcomes
☒	☐	Estimates of effect sizes (e.g. Cohen's *d*, Pearson's *r*), indicating how they were calculated

Our web collection on statistics for biologists contains articles on many of the points above.

Software and code

Policy information about availability of computer code

Data collection	Leica LAS X and Zeiss ZEN imaging sortware was used for imaging.
Data analysis	Data were analyzed using Fiji.

For manuscripts utilizing custom algorithms or software that are central to the research but not yet described in published literature, software must be made available to editors/reviewers. We strongly encourage code deposition in a community repository (e.g. GitHub). See the Nature Research guidelines for submitting code & software for further information.

Data

Policy information about availability of data

All manuscripts must include a data availability statement. This statement should provide the following information, where applicable:

- Accession codes, unique identifiers, or web links for publicly available datasets
- A list of figures that have associated raw data
- A description of any restrictions on data availability

Provide your data availability statement here.

Field-specific reporting

Please select the one below that is the best fit for your research. If you are not sure, read the appropriate sections before making your selection.

☒ Life sciences ☐ Behavioural & social sciences ☐ Ecological, evolutionary & environmental sciences

For a reference copy of the document with all sections, see nature.com/documents/nr-reporting-summary-flat.pdf

Life sciences study design

All studies must disclose on these points even when the disclosure is negative.

Sample size	No sample size calculation was performed. The chosen sample sizes are documented in the related Source Data file for all quantifications.
Data exclusions	No data have been excluded from any analysis.
Replication	All experiments have been repeated at least three times independently.
Randomization	No randomization was required for the presented data.
Blinding	*Describe whether the investigators were blinded to group allocation during data collection and/or analysis. If blinding was not possible, describe why OR explain why blinding was not relevant to your study.*

Reporting for specific materials, systems and methods

We require information from authors about some types of materials, experimental systems and methods used in many studies. Here, indicate whether each material, system or method listed is relevant to your study. If you are not sure if a list item applies to your research, read the appropriate section before selecting a response.

Materials & experimental systems

n/a	Involved in the study	
☐	☒	Antibodies
☐	☒	Eukaryotic cell lines
☒	☐	Palaeontology
☒	☐	Animals and other organisms
☒	☐	Human research participants
☒	☐	Clinical data

Methods

n/a	Involved in the study	
☒	☐	ChIP-seq
☒	☐	Flow cytometry
☒	☐	MRI-based neuroimaging

Antibodies

Antibodies used	anti-HA (Santa Cruz, sc-7392), anti-β-actin (Invitrogen, MA1-140), anti-LAMP2 (Santa Cruz, sc-18822), anti-LAMP1 (CST, 9091), anti-LC3 (MBL International, M152-3), anti-p62 (BD Biosciences, 610833), anti-STX17 (GeneTex, GTX130212), anti-EEA1 (CST, 3288), anti-Rab5 (CST, 3547), anti-Rab7 (CST, 9367), anti-Rab11A (Invitrogen, 700184), anti-Ub (Enzo Life Sciences, BML-PW8810), anti-Hsp60 (CST, 12165), anti-PMP70 (Sigma-Aldrich, SAB4200181), anti-EGFR (Santa Cruz, sc-03-G), anti-PtdIns(4)P (Echelon, Z-P004), anti-GFP (Invitrogen, A-6455), anti-myc (9E10, Biochemistry core facility, MPI of Biochemistry, Martinsried), anti-PtdIns(4,5)P2 (Echelon, Z-P045).
Validation	All antibodies were validated by the vendors and documented by corresponding data sheets that can be requested from those vendors using the information above. The anti-myc antibody was validated by the Biochemistry core facilty of the MPI of Biochemestry and documantation will be made available upon request.

Eukaryotic cell lines

Policy information about cell lines

Cell line source(s)	HeLa cells from Cell Lines Service (# 300194) and human neural stem cells from Thermo Fisher scientific (# N7800) have been used.
Authentication	Authentication was performed by the vendors and corresponding information can be requested from them.
Mycoplasma contamination	All cells were tested for Mycoplasm contaminations on a regular basis using PCR methods. No contamination was detected.
Commonly misidentified lines (See ICLAC register)	N.A.
