# Supplementary material for: TECPR1 promotes aggrephagy by direct recruitment of LC3C autophagosomes to lysosomes
Source: Nat Commun. 2020 Jun 12;11:2993. doi: 10.1038/s41467-020-16689-5 (PMC7293217; doi:10.1038/s41467-020-16689-5)
Supplement: Supplementary file 2 — Peer Review File [file 41467_2020_16689_MOESM2_ESM.docx]

Peer Review Information

**Journal:** Nature Communications

**Manuscript title:** TECPR1 promotes aggrephagy by direct recruitment of LC3C autophagosomes to lysosomes

**Corresponding author name(s):** Dr Thomas Wollert

**Editorial notes:**

**Transferred manuscripts**

This manuscript has been previously reviewed at another journal that is not operating a transparent peer review scheme. This document only contains reviewer comments and rebuttal letters for versions considered at *Nature Communications*.

**Transferred manuscripts (no peer review at *Nature Communications*)**

This manuscript has been previously reviewed at another journal that is not operating a transparent peer review scheme. The manuscript was considered suitable for publication without further review at *Nature Communications*.

**Redactions – transferred manuscripts (mention of previous referee reports from elsewhere)**

This manuscript has been previously reviewed at another journal that is not operating a transparent peer review scheme. This document only contains reviewer comments and rebuttal letters for versions considered at *Nature Communications*. Mentions of prior referee reports have been redacted.

**Redactions – transferred manuscripts (mention of the other journal)**

This manuscript has been previously reviewed at another journal that is not operating a transparent peer review scheme. This document only contains reviewer comments and rebuttal letters for versions considered at *Nature Communications*. Mentions of the other journal have been redacted.

**Redactions – unpublished data**

Parts of this Peer Review File have been redacted as indicated to maintain the confidentiality of unpublished data.

**Redactions – confidential patient information**

Parts of this Peer Review File have been redacted as indicated to maintain patient confidentiality.

**Redactions – published data**

Parts of this Peer Review File have been redacted as indicated to remove third-party material where no permission to publish could be obtained.

**Amendments – published data**

[Figure X/The figure on page X] in this Peer Review File has been amended to remove third-party material where no permission to publish could be obtained.

**Published data (credit line for image)**

[Figure X/The figure on page X] in this Peer Review File is reproduced with permission from [Copyright Holder].

**Redactions – reviewer opt-out**

Parts of this Peer Review File have been redacted as indicated as we could not obtain permission to publish the reports of reviewer ###.

**Reviewer comments in marked-up manuscript**

In their review of the [first/second/third/...] version of this manuscript, reviewer ### added their comments to the manuscript file. These comments, excluding minor textual revisions, have been copied into this Peer Review File.

**Reviewer comments & decisions:**

**Reviewer comments, first version:**

Reviewer #1 (Remarks to the Author):

This study provides evidence that TECPR1 assists autophagosome-lysosome fusion by targeting LC3C to lysosomes. The model proposed is new and challenges what was proposed by Qing Zhing’s lab in their Mol Cell paper. The paper includes some nice strategies (e.g. targeting to TECPR1 to endosomes enables powerful experiments– Fig 5c). The data showing that TECPR1 is involved in selective autophagy, or clearance of puromycin-induced aggregates are not novel and have been published before (Ogawa et al Cell Host Microbe. 2011 May 19;9(5):376-89).

Major comments:
Many of the experiments use proximity based approaches to infer interactions – but the key experiments using immunoprecipitations from cells have not been presented. This is critical for the experiments testing interactions (e.g. LC3C with TECPR1, including the relevant mutants ) as BIoID hits may not be direct. The in vitro experiments are not sufficient to make the point either.
It may be useful to assess TECPR1 localisation in cells where LC3 containing autophagosomes are ablated - e.g. using ATG5 or ATG12 knockdowns.
Does TECPR1 reduce protein aggregates in cells where LC3C is knocked down?
Does LC3C knockdown affect clearance of protein aggregates?


Reviewer #2 (Remarks to the Author):

I'm not anywhere close to being comfortable judging the biological merits of the manuscript. However I do believe that the structured-illumination images obtained with Zeiss Elyra are of high quality and do support the conclusions drawn.

There's maybe a small mistake in the 2nd paragraph on Page 7: "GFP-LC3C was, however, found to form much smaller puncta, which were not colocalizing with but in close proximity to GFP-TECPR1 puncta". Perhaps you meant "RFP-TECPR1"?


Reviewer #3 (Remarks to the Author):

TECPR1 has been shown to promote autophagy in the autolysosome step by associating with Atg5. Here the authors did more work to reveal the mechanism regarding how TECPR1 promotes autophagosome maturation. They found that that TECPR1 specifically associates with LC3C and PI4P. The association between LC3C and TECPR1 may promote the clearance of ubiquitin and aggregates. While the specific association between TECPR1 and LC3C looks interesting, the overall data is not clear enough to support the claims made. More improvements are necessary.
Major points:
1. All the imaging data are based on overexpression of GFP tagged proteins which may cause significant artifact endogenous proteins should be stained and analyzed to confirm.
2. The pictures the authors selected as well as the quantification bars are not consistent between figures. These make the reviewer worried about the overall quality of data and how representative they are.
For example: an obvious inconsistency could be found in Fig1c and d. Where 1c indicates an average of 50 LC3c puncta per cell, but 1d only shows a couple of LC3C puncta. Therefore the picture is not representative.
3. Almost all conclusions are based on counting puncta. The work lacks biochemical data to confirm the imaging data. For example, degradation of P62 and Ub in Fig7 should be determined by WB to confirm the imaging data. Another example is the lack of proteinase K protection assay to claim that LC3C localize on the outer membrane of the autophagosome.
4. The author could not explain why LC3C over the others selectively associates TECPR1. Although a non-selective LIR is involved, but it only partially accounts for LC3C-TECPR1 association based on Fig.2d. Later the author found that deletion of the LIR totally abolished TECPR1 with LC3C in Fig S5. These are inconsistent results. One explanation would be the misuse of the LC3 antibody (M152-3 MBL) which only recognizes LC3A/B but misused as a pan LC3 antibody in Fig.S5.
5. Potential data misinterpretation. For example: Fig.7e, the authors claim that LC3B localizes inside of LC3C. However, the small LC3B puncta that looks inside of LC3C could also be an artifact of weak LC3B signal that leads to artificial smaller puncta. Another example, in Fig.S6, the authors claim specificity of TECPR1 for P62 over peroxisome or mitochondria. However, the PMP70 stain looks not working well. It is not prudent to draw conclusions based on poor antibody staining.
Other points:
1. The MYC blot in Fig1a input is not clear enough to me. It is hard to image the authors could obtain a sharp band after IP based on the blurry input. Please improve.
2. Quantification and sufficient labeling should be included for movies. For example, how many structures are dynamic and how dynamic are they? For the dynamic small structures and stationary big ones what is the size difference? In the movie/picture, which are the dynamic and which are the stationary? Please label clearly.
3. The EM image in Fig.S1 e and f is distracting and does not add much to the work. The author could consider removing them.
4. In Fig.4d, the reviewer is not convinced that the TECPR1 and PI4P doubly labeled area colocalize with lamp2.


Reviewer #4 (Remarks to the Author):

The manuscript by Dempfle et al reports that the protein TECPR1, previously implicated in selective autophagy of bacteria, interacts with the autophagosomal protein LC3C and may be important for recruiting LC3C-containing autophagosomes to lysosomes. This functional interaction is examined in the context of selective removal of aggregates in neuronal stem cells.
The work is of strong interest and the experiments reported are, for the most part, supportive of the main proposal.
The biochemical characterization of the TECPR1-LC3C interaction and the possibility that the PH domain of TECPR1 binds PI4P in vitro as a potential mechanism of lysosome recruitment are convincing.
I was less convinced that this interaction, and especially a tripartite one between TECPR1/PI4P/lysosomes is strong in vivo (Fig 4 C,D) because the co-localization is low and there are no experiments specifically manipulating the PI4P pool.
The least convincing aspect was the effects of TECPR1 on aggrephagy in neuronal stem cells. Here I was puzzled by the absence of any co-localization between ubiquitin signals and TECPR1 (Fig 7 D). I am also interested in the possibility that the presence of the 2X-FYVE proteins induces ubiquitin response on its own, as opposed to the wild type clearing the aggregates. Finally, this type of phenotype, which involves TECPR1-clustered autophagosomes attacking protein aggregates, must be examined by some type of CLEM.
Of minor concern:
-What happens to this system during starvation?
-It would be good to show some more experiments with endogenous LC3 upon TECPR1 overexpression at the beginning
-Is the TECPR1 construct missing the PH domain less punctate (Fig 4)?
-Why is TECPR1 vacuolar in the RFP-2xFYVE overexpressing cells (Fig S4)?
-Does TECPR1 overexpression inhibit PI(4,5)P2 levels? In some of the photos it would appear to be so.

**Author rebuttal, first version:**

Reviewer #1 (Remarks to the Author):

This study provides evidence that TECPR1 assists autophagosome-lysosome fusion by targeting LC3C to lysosomes. The model proposed is new and challenges what was proposed by Qing Zhing’s lab in their Mol Cell paper. The paper includes some nice strategies (e.g. targeting to TECPR1 to endosomes enables powerful experiments– Fig 5c). The data showing that TECPR1 is involved in selective autophagy, or clearance of puromycin-induced aggregates are not novel and have been published before (Ogawa et al Cell Host Microbe. 2011 May 19;9(5):376-89).

We thank this reviewer for appreciating our strategy to investigate TECPR1 function by engineering an endosomal targeted variant. We also agree that some aspects of our work have been reported previously. However, the unique findings in our study concern the molecular mechanism involved in targeting proteins aggregates to lysosomal degradation by autophagy. Our results show that LC3C is recruited to protein aggregate containing autophagosomes and that the interaction of LC3C and TECPR1 is important for their targeting to lysosomes for degradation. Moreover, the strategy of targeting TECPR1 to early endosomes revealed that TECPR1 dictates the itinerary of LC3C-positive autophagosomes. Thus, although the fusion reaction itself depends on and is orchestrated by many other factors, including SNAREs and membrane tethers, TECPR1 acts most upstream.

Major comments:
Many of the experiments use proximity based approaches to infer interactions – but the key experiments using immunoprecipitations from cells have not been presented. This is critical for the experiments testing interactions (e.g. LC3C with TECPR1, including the relevant mutants ) as BIoID hits may not be direct. The in vitro experiments are not sufficient to make the point either.

We entirely agree that Bio-ID experiments are only revealing spatial vicinity of molecules, but not their direct interaction. As requested by the reviewer, we have performed co-immunoprecipitation experiments using GFP-TECPR1 or GFP as bait. Consistent with our in vitro data, we found that only GFP-TECPR1 but not GFP alone co-immunoprecipitated HA-LC3C. We included this new data now in the manuscript as ***new Figure 1b***.

It may be useful to assess TECPR1 localisation in cells where LC3 containing autophagosomes are ablated - e.g. using ATG5 or ATG12 knockdowns.

We agree that investigating the localization of TECPR1 in cells in which autophagy is inhibited will provide interesting information whether or not the localization of TECPR1 depends on autophagy. We therefore treated cells with the autophagy inhibitor wortmannin and investigated the localization of TECPR1 as well as TECPR1∆PH-2xFYVE (Reviewer Figure 1). Due to the non-selective inhibition of all PI3-kinases by wortmannin, we assumed that the localization of TECPR1∆PH-2xFYVE would be wortmannin-sensitive, while that of TECPR1 would not, provided that the recruitment of TECPR1 to lysosomes is independent of autophagy and PI3P.

**Reviewer Figure 1: Localization of TECPR1 in cells in which autophagy was inhibited.** Neural stem cells were transfected with GFP-TECPR1 or GFP-TECPR1∆PH-2xFYVE and autophagy was inhibited by treatment with wortmannin for 4 hours. Protein aggregation was induced by puromycin treatment to increase the number of protein aggregates. Cells were fixed and immunostained for LAMP2, EEA1, Ub, or LC3C using respective antibodies (for details see Methods section in the manuscript). (**A**) Inhibition of autophagy did not impact on the colocalization of TECRP1 to lysosomes. (**B**) Interestingly, GFP-TECPR1∆PH-2xFYVE is not redirected to early endosomes if PI3P is depleted from early endosomes by wortmannin.

We indeed observed that the colocalization of TECPR1 with the lysosomal marker LAMP2 was independ of wortmannin. By contrast, a strongly enhanced colocalization of TECPR1∆PH-2xFYVE with LAMP2 and a markedly reduced colocalization with EEA1 was observed in TECPR1∆PH-2xFYVE expressing cells that have been treated with wortmannin.

Does TECPR1 reduce protein aggregates in cells where LC3C is knocked down?
Does LC3C knockdown affect clearance of protein aggregates?

To answer these two questions, we performed a comprehensive series of pulse-chase experiments to follow clearance of puromycin induced protein aggregates. We treated cells first with siRNAs for indicated times, followed by puromycin treatment for two to four hours (pulse) and chased the clearance of protein aggregates upon puromycin retrieval. The results are summarized in **Reviewer Figure 2**.

**Reviewer Figure 2: Protein aggregate clearance assays.** (**A**) TECPR1 wt and TECPR1 KO cells were treated with siRNAs as indicated for 24 ours followed by treatment with puromycin for 2 hours. The clearance of protein aggregates was followed by a chasing phase for 2 hours during which proteasomal degradation of proteins was blocked by addition of MG132. Autophagic degradation of protein aggregates is impaired in TECPR1 KO cells, upon knockdown of LC3C, and if LC3C is knocked down in TECPR1 KO cells. (**B**) HeLa cells were treated with siRNAs as indicated for 72 h and incubated with puromycin for 4 hours followed by a 4 hour chasing phase. Autophagic degradation of protein aggregates is strongly impaired in siGABARAP cells. (**C**) HeLa cells were treated with siRNA against GABARAPs or control siRNA and transfected with GFP-TECPR1∆PH-2xFYVE. Fixed cells were immunostained with a nonselective LC3 antibody and an EEA1 antibody. A significant accumulation of LC3 puncta at EEA1-positive GFP-TECPR1∆PH-2xFYVE structures was observed if GABARAPs were knocked down. (**D**) Quantification of the colocalization of LC3 positive structures with TECPR1 and EEA1 from images as shown in **C** using the Pearson coefficient.

Liu et al. (2017) previously reported that knockdown of LC3C induces accumulation of protein aggregates. Consistent with this report, we observed a strong reduction of the clearance of protein aggregates if LC3C was knocked down in wildtype cells. Moreover, the clearance of protein aggregates was also impaired if TECPR1 was knocked out. The strongest impairment of protein clearance was observed in TECPR1 KO cells that were treated with siLC3C. This suggests that TECPR1 and LC3C act synergistically (Reviewer Figure 2A). Previous studies reported that the combined knockout of GABARAP proteins impacts on the fusion of autophagosomes with lysosomes, while that of LC3 proteins did not. We therefore tested protein aggregate clearance in cells in which all GABARAP family members were knocked down using siRNAs. We indeed observed a strong delay in aggregate clearance if all GABARAP proteins were depleted (**Reviewer Figure 2B**). Furthermore, we found that accumulated protein aggregates were redirected to EEA1 positive structures in GABARAP KD cells that expressed TECPR1∆PH-2xFYVE (**Reviewer Figure 2C** and **D**). These data suggest that although GABARAPs are important for the fusion of autophagosomes with lysosomes, they are dispensable for the LC3C dependent recruitment of protein aggregate containing autophagosomes by TECPR1.

Reviewer #2 (Remarks to the Author):

I'm not anywhere close to being comfortable judging the biological merits of the manuscript. However I do believe that the structured-illumination images obtained with Zeiss Elyra are of high quality and do support the conclusions drawn.

We thank the reviewer for evaluating our super-resolution data. We particularly appreciate, that the reviewer acknowledged our efforts to perform high resolution imaging using the Zeiss Elyra and for judging our data to be of high quality.

There's maybe a small mistake in the 2nd paragraph on Page 7: "GFP-LC3C was, however, found to form much smaller puncta, which were not colocalizing with but in close proximity to GFP-TECPR1 puncta". Perhaps you meant "RFP-TECPR1"?

We thank the reviewer for alerting us regarding this mistake and have corrected it.

Reviewer #3 (Remarks to the Author):

TECPR1 has been shown to promote autophagy in the autolysosome step by associating with Atg5. Here the authors did more work to reveal the mechanism regarding how TECPR1 promotes autophagosome maturation. They found that that TECPR1 specifically associates with LC3C and PI4P. The association between LC3C and TECPR1 may promote the clearance of ubiquitin and aggregates. While the specific association between TECPR1 and LC3C looks interesting, the overall data is not clear enough to support the claims made. More improvements are necessary.

We thank the reviewer for evaluating our manuscript and for acknowledging our new mechanistic insights into the function of TECPR1. Moreover, we highly appreciate the valuable comments of this reviewer. Thanks to these suggestions, we performed many additional experiments as outlined in our point-by-point response to directly address the concerns of this reviewer.

Major points:
1. All the imaging data are based on overexpression of GFP tagged proteins which may cause significant artifact endogenous proteins should be stained and analyzed to confirm.

We entirely agree with the reviewer that data on endogenous proteins could strengthen our findings. We indeed dedicated a significant amount of time and effort to set up experiments in which we could specifically stain the respective endogenous proteins. For TECPR1, for example, we performed pull-down and immunofluorescence experiments using commercially available antibodies (Santa Cruz sc-240971, Cell Signaling Technology cat. no. 8097). Furthermore, we tested all available antibodies that are supposed to be LC3C selective. However, none of the tested TECPR1 antibodies showed specific staining (Santa Cruz antibody was already retracted by the vendor) and LC3C antibodies showed strong cross-reactivity towards LC3A and LC3B. Due to the fact that we recombinantly expressed and purified all corresponding proteins, we could use these proteins as positive controls in western blots to identify the corresponding bands. We are therefore very certain that both TECPR1 and LC3C antibodies are not sufficiently selective.

To address the concern of the reviewer that overexpression induces adverse effects that could influence the conclusion of our study, notably that LC3C is specifically recruited to autophagosomes that contain protein aggregates, we performed experiments in which we blocked autophagy. We therefore treated neural stem cells that overexpressed LC3C with the autophagy inhibitor wortmannin. We observed that wortmannin inhibits the formation of LC3C puncta, confirming that (i) LC3C is not aggregating itself if overexpressed and (ii) the formation of LC3C puncta depends on autophagy. The corresponding data of these experiments are now presented in **Reviewer Figure 3**.

**Reviewer Figure 3: Formation of LC3C puncta depends on autophagy.** Autophagy was inhibited using the inhibitor of the PI3-kinase complex wortmannin at a final concentration of 150 nM. Neural stem cells were transfected with GFP-TECPR1 and RFP-LC3C constructs and treated with the inhibitor for 4 hours (bottom panel). Non-treated cells served as control (upper panel).

2. The pictures the authors selected as well as the quantification bars are not consistent between figures. These make the reviewer worried about the overall quality of data and how representative they are.
For example: an obvious inconsistency could be found in Fig1c and d. Where 1c indicates an average of 50 LC3c puncta per cell, but 1d only shows a couple of LC3C puncta. Therefore the picture is not representative.

We thank the reviewer for this comment and for critically analyzing our data. In Figure 1d one confocal slice of the cell is shown, covering only 10 % of its total volume. Still, apart from the big obvious structure, additional smaller puncta can be observed. If one takes only very significant puncta above background, there are 8 puncta only in this confocal section. So in total the cell would contain many more. We performed the data analysis in an unbiased way by applying an Fiji-based algorithm the takes data from z-stacks, reconstructs a three-dimensional volume, subtracts background, and counts puncta. We defined puncta using fluorescence with an intensity that was significantly higher as the background fluorescence and which had intrinsic symmetry. ***This approach has the great advantage that all data presented in the manuscript are unbiased counts with the identical measures to decide what a punctum is***. We have developed the corresponding algorithm with image-analysis experts that are specialized for fluorescence microscopy data. We are therefore sure and certain, that the images we are showing as confocal slice and the data from quantifications of reconstructed entire cell volumes meet highest standards.

3. Almost all conclusions are based on counting puncta. The work lacks biochemical data to confirm the imaging data. For example, degradation of P62 and Ub in Fig7 should be determined by WB to confirm the imaging data. Another example is the lack of proteinase K protection assay to claim that LC3C localize on the outer membrane of the autophagosome.

We thank the reviewer for this comment and agree that our initial manuscript contained many data that were based on quantification of puncta in confocal images. We performed additional biochemical experiments as requested by the reviewer. The requested protease K protection assay has been performed and the corresponding data are now included as new **Figure 6d** in our revised manuscript. We also performed a quantification of these data and found that 60% of GFP-LC3C is degraded by proteinase K in the absence and 90% in the presence of detergent. Thus, the majority of LC3C is located outside of autophagosomes, while a small fraction of 30% is protected by proteinase K and thus located inside membrane vesicles/autophagosomes. The localization of a portion of LC3C inside vesicles is consistent with a function of LC3C as cargo tether in selective autophagy.

We extended our biochemical characterization by performing immunoprecipitation experiments of GFP-TECPR1 and HA-LC3C expressing cells to confirm that both proteins interact with each other. The results of these experiments are shown in our new **Figure 1b**.

Regarding the degradation of p62 and Ub in Fig7, we performed western blots of neural stem cells that expressed TECPR1 or TECPR1∆PH-2xFYVE in the presence and absence of Bafilomycin A. We, however, encountered technically extremely challenging problems that prevented us from obtaining reliable data. The transfection efficiency of neural stem cells is approximately 10%. Thus, given that very few or no protein aggregates are present in transfected cells, the reduction of total protein aggregates in cell lysates of the mixed population of non-transfected and transfected cells would therefore not exceed 10%. A maximal difference of Ub or p62 levels between lysates of non-transfected and transfected cells of 10% could therefore be expected, which we could not reliably detect although some tendencies were observed. To avoid influence from high background level of non-transfected cells, we next established cell sorting of NSCs to enrich the population of transfected cells. To obtain enough cells after sorting for western blot analysis (10^6^ per condition), we needed to grow approximately 2x10^7^ cells. During expansion of NPCs to reach these large numbers, the cells started to undergo morphological changes indicating that they differentiate. The status of these cells was thus not well defined. Moreover, many cells were damaged or ruptured during the sorting, which liberated proteases and let to a significant reduction in total protein amounts that we recovered from sorted cells. We tried to optimize the conditions but did not reach satisfying sample quantity. We invested an enormous amount of recourses including apart from numerous working hours of two team members 5000 € for transfection kits and 3100 € for medium. For all these reasons, we could not continue to try to establish condition that allowed us to perform these experiments.

In order to support our claims that protein aggregates are cleared in NSC that overexpressed TECPR1, we applied an alternative approach. We performed live cell imaging and followed fusion of TECPR1 positive compartments with LC3C positive puncta (Supporting Movies 7, 8, 9 and 10). We therefore recorded z-stacks of cells expressing RFP-LC3C and either GFP-TECPR1 or GFP-TECPR1∆PH-2xFYVE and followed their interaction over time in 3D reconstructed cell volumes. We observed fusion of puncta in TECPR1 expressing cells. Interestingly, we often observed fusion of LC3C puncta with TECPR1 structures that already colocalized with RFP, indicating that TECPR1 structures receive more than one LC3C structures. This is consistent with our observation that TECPR1 localizes to autolysosomes (see our CLEM data from Fig. S1e). By contrast, LC3C puncta associated over long times with TECPR1∆PH-2xFYVE structures without fusion.

4. The author could not explain why LC3C over the others selectively associates TECPR1. Although a non-selective LIR is involved, but it only partially accounts for LC3C-TECPR1 association based on Fig.2d. Later the author found that deletion of the LIR totally abolished TECPR1 with LC3C in Fig S5. These are inconsistent results. One explanation would be the misuse of the LC3 antibody (M152-3 MBL) which only recognizes LC3A/B but misused as a pan LC3 antibody in Fig.S5.

We entirely agree with the reviewer that that LIR motif is not sufficient to provide selectivity for LC3C. This has been shown, as the reviewer was referring to, in vitro, using the LIR peptide and LUVs to which LC3 variants were conjugated and in vivo using BioID experiments (Fig 2d). Concerning the interaction of TECPR1∆PH-2xFYVE in which the LIR motif has been mutated, we still observed a colocalization of LC3 at TECPR1 variants W175A, I178A and W175A/I178A, although the number of puncta is strongly reduced (Fig. 5e). This corresponds very well with the reduced, but not entirely abolished biotinylation of LC3C in BioID tagged TECPR1-LIR mutant expressing cells.

The specificity of the used LC3 antibody is much broader than indicated by the vendor. We performed a series of experiments to carefully evaluate its specificity. We first used our recombinant proteins and found that the antibody M152-3 MBL detects LC3A, B, and C with similar efficiencies in western blot analysis. We confirmed these results by detecting LC3 proteins in western blots of lysates from cells that overexpressed one of the LC3 proteins. We next tested the colocalization of GFP-LC3C and LC3 by immunofluorescence using the antibody M152-3 MBL and found that all GFP-positive puncta colocalized with puncta stained by that antibody. Recognition of LC3C by M152-3 MBL is not surprising given that the entire LC3B protein (residues 1-120) has been used as immunogen and most of the residues of LC3 proteins are identical. The only significant variation includes the N-terminal helix of LC3 proteins, in which considerable differences between LC3A, B and C are present.

5. Potential data misinterpretation. For example: Fig.7e, the authors claim that LC3B localizes inside of LC3C. However, the small LC3B puncta that looks inside of LC3C could also be an artifact of weak LC3B signal that leads to artificial smaller puncta.

We agree that from this data a potential localization of LC3B cannot be inferred. We removed the statement and thank the reviewer for this important comment.

Another example, in Fig.S6, the authors claim specificity of TECPR1 for P62 over peroxisome or mitochondria. However, the PMP70 stain looks not working well. It is not prudent to draw conclusions based on poor antibody staining.

# We thank the reviewer for indicating that the staining of PMP70 is not sufficient to draw any conclusion. We now repeated the immunostaining experiment using an optimized staining protocol. The data are now presented in the new Figure S6a. From this analysis it is very obvious that peroxisomes and LC3C are not colocalizing.

Other points:
1. The MYC blot in Fig1a input is not clear enough to me. It is hard to image the authors could obtain a sharp band after IP based on the blurry input. Please improve.

This comment of the reviewer relates to the Myc signal in the input of TECPR1-BioID-myc. The problem with detecting TECPR1-BioID-myc in cell lysates is mainly related to its size (180 kDa), rendering its transfer from gels to the blotting membrane inefficient. Moreover, the myc antibody has particularly in lysates a higher background. After pull-down, TECPR1 is enriched through a residual interaction of the BioID protein with biotin. Consequently, the detection even after inefficient transfer to the blotting membrane is much better, resulting in a much better signal to noise ratio. The relevant information from the TECPR1 blot of cell lysates is, that TECPR1-BioID-myc is expressed in all samples.

2. Quantification and sufficient labeling should be included for movies. For example, how many structures are dynamic and how dynamic are they? For the dynamic small structures and stationary big ones what is the size difference? In the movie/picture, which are the dynamic and which are the stationary? Please label clearly.

# We thank the reviewer for indicating that the labeling of our movies should be improved. We have annotated all movies and provided explanations of the annotations in the corresponding movie legends. Furtherore, we performed the quantifications as requested be the reviewer.

# Movie S2: TECPR1 structures: 16 ± 2 % of structures are large (>1.0 µm) and slow, the remaining structures are small (usually <0.5 µm) and fast. Labels were included in the movie. The movie has been annotated with an arrowhead.

# Movie S3: 25 % of lysotracker positive structures colocalize with TECPR1, theses lysosomes appear to be larger (>1 µm) and less mobile than the smaller structures that are TECPR1 negative. The circular TECPR1 structures that surrounds a lysotracker puncta is labeled with an arrowhead.

# Movie S4: The majority of LC3C puncta is small (85%, < 0.5 µm) and mobile. A LC3C puncta that traffics towards and associates with a lysotracker positive structure (lysotracker structures are < 1 µm in size and immobile) is indicted by an arrowhead.

3. The EM image in Fig.S1 e and f is distracting and does not add much to the work. The author could consider removing them.

We agree that these data are supportive but not essential to support the major conclusion of the manuscript. However, we do think that it is interesting to present these data as supplemental material since these CLEM data confirm not only previous observations, but demonstrates that TECPR1 forms two different pools on membranes in vivo, i.e. an autolysosomal and a vesicular pool.

4. In Fig.4d, the reviewer is not convinced that the TECPR1 and PI4P doubly labeled area colocalize with lamp2.

The reviewer is right that there are two different observations. LAMP1 colocalizes with TECPR1 as seen in many images. In Figure 4d, we used structured illumination microscopy which allows a much better resolution of the fluorescence signals. LAMP1 positive lysosomal membranes appear indeed to be spatially separated from PI4P membranes, indicating the lysosomes possess co-existing LAMP1 and PI4P domains. TECPR1 localizes specifically to PI4P domains if the PH domain is present, but not if the PH domain is deleted. The apparent distraction thus comes from the higher resolution of SIM data, which reviewer 2 was describing as: “*structured-illumination images obtained with Zeiss Elyra are of high quality and do support the conclusions drawn”.*

Reviewer #4 (Remarks to the Author):

The manuscript by Dempfle et al reports that the protein TECPR1, previously implicated in selective autophagy of bacteria, interacts with the autophagosomal protein LC3C and may be important for recruiting LC3C-containing autophagosomes to lysosomes. This functional interaction is examined in the context of selective removal of aggregates in neuronal stem cells.
The work is of strong interest and the experiments reported are, for the most part, supportive of the main proposal.

We highly appreciate the time and effort of this reviewer in evaluating our manuscript. We furthermore thank for the very valuable suggestion how to improve the manuscript and in identifying data that need to be improved or complemented by additional experiments. As this reviewer will see from our point-by-point response, we took all points very seriously and are happy to provide for all concerns of this reviewer new data.

The biochemical characterization of the TECPR1-LC3C interaction and the possibility that the PH domain of TECPR1 binds PI4P in vitro as a potential mechanism of lysosome recruitment are convincing.

We thank this reviewer for acknowledging the quality of our data concerning the function of the PH domain of TECPR1.

I was less convinced that this interaction, and especially a tripartite one between TECPR1/PI4P/lysosomes is strong in vivo (Fig 4 C,D) because the co-localization is low and there are no experiments specifically manipulating the PI4P pool.

We entirely agree with this reviewer. We are convinced that there is no tripartite interaction of TECPR1, LAMP1 and PI4P. We observed a strong colocalization of LAMP1 and TECPR1 in confocal microscopy. However, using structured illumination microscopy with much better resolution revealed that LAMP1 domains and TECPR1/PI4P domains are separated. On the other hand, our CLEM data (Figure S1e) support the lysosomal localization of TECPR1. We therefore suggested in our manuscript that there might be a co-existence of TECPR1/PI4P and LAMP1 domains on lysosomes. This notion is supported by our observation that in the absence of the PH-domain, the lysosomal localization of TECPR1 is retained (Fig. 4A) but its colocalization with PI4P is lost (Fig. 4c).

To support these claims, we performed additional experiments as suggested by this reviewer and manipulated the PI4P pool in cells by knock-down of the PI4K IIα, that has been shown to be important for autophagosome-lysosome fusion previously, as well as overexpression of the PI4P-phosphatase Sac2 as shown in Reviewer Figure 4.

**Reviewer Fig.4: Manipulation of the PI4P pool in HeLa cells and its impact on the lysosomal localization of TECPR1.** (A) The colocalization of TECPR1 and LAMP2 was investigated in HeLa cells that have been treated with siRNAs against PI4K ii α (Phosphatidylinositol(4)-kinase 2 alpha) and OCRL (Phosphatidylinositol(5)-phosphatase) or both. (B) Colocalization of PI4P and TECPR1 in HeLa cells treated with siRNA against PI4K II α. (C) Colocalization of TECPR1, Sac2 and TECPR1 in HeLa cells.

Our new data support nicely our major conclusion that the PH-domain of TECPR1 is important for its co-localization with PI4P, but not for its lysosomal targeting. This supports our observation that the TR1 domain of TECPR1 is required and sufficient for lysosomal localization, but not for PI4P binding. We could demonstrate with our additional experiments that the knock-down of PI4K by siRNA leads to a depletion of PI4P (compare PI4P specific staining in control and PI4K knock-down cells (Reviewer Fig. 4B). While this depletion also reduces the colocalization of TECPR1 with PI4P, its lysosomal targeting is unaffected. Moreover, knock-down of the Phosphatidylinositol(5)-phosphatase OCRL (an enzyme that restores PI4P levels on lysosomes by converting PI4,5P2 into PI4P), has also no impact on the colocalization of TECPR1 and the lysosomal marker LAMP2. Finally, the Phosphatidylinositol(4)-phosphatase Sac2 co-localizes with TECPR1 and LC3C, suggesting that PI4P is dephosphorylated at TECPR1/PI4P domains and thus modulates or restricts such domains, without impacting on the recruitment of LC3C to lysosomes.

The least convincing aspect was the effects of TECPR1 on aggrephagy in neuronal stem cells. Here I was puzzled by the absence of any co-localization between ubiquitin signals and TECPR1 (Fig 7 D).

We thank the reviewer for this comment. We were also surprised to see a segregation of Ub and TECPR1 signals in BafA1 treated cells. In puromycin treated neural stem cells that were not treated with BafA1, we observed a strong colocalization of LC3C and Ub, whereas the colocalization of LC3C and Ub in BafA1 treated cells was much weaker (Figure S7c). This indicates that the treatment with BafA1 impacts on the recruitment of LC3C to Ub-puncta. As a consequence, such Ub puncta are not recruited to TECPR1 structures.

To confirm that expression of TECPR1 leads to a rapid degradation of LC3C-positive structures we now performed new experiments in which we followed trafficking of RFP-LC3C puncta in GFP-TECPR1 expressing neural stem cells over time. We found that RFP-LC3C puncta are trafficking to TECPR1 positive structures followed by fusion of these structures. The corresponding data are shown as Supplemental Movies 7 and 8, that are now part of the manuscript. Interestingly, if we expressed instead of TECPR1 the mutant TECPR1∆PH2xFYVE together with RFP-LC3C, we observed that TECPR1 structures colocalized with LC3C puncta over long times without fusion.

I am also interested in the possibility that the presence of the 2X-FYVE proteins induces ubiquitin response on its own, as opposed to the wild type clearing the aggregates.

This is indeed a very interesting question and we thank this reviewer for bringing it up. In our initial manuscript we reported that inhibition of autophagy by BafA1 treatment prevents clearance of protein aggregates in TECPR1 transfected cells, arguing that overexpression of TECPR1 leads first to an accumulation of protein aggregates, which are, however, rapidly cleared in an autophagy dependent fashion. To demonstrate, that protein aggregates are re-routed to early endosomes in TECPR1∆PH-2xFYVE expressing NSCs, we transfected cells with GFP-TECPR1∆PH-2xFYVE and immunostained for Ubiquitin and EEA1 as shown in Reviewer Figure 5.

**Reviewer Figure 5: Formation of ubiquitin clusters at early endosomes by TECPR1∆PH-2xFYVE.** HeLa cells, transfected with GFP-TECPR1∆PH-2xFYVE and immuno-stained for Ubiquitin and EEA1 are shown.

As already presented in the initial manuscript, we observed an accumulation of Ubiquitin at EEA1 positive structures in cells expressing TECPR1∆PH-2xFYVE. Thus, if TEPCR1 is rerouted away from lysosomes to endosomes, ubiquitinated protein aggregates are no longer cleared through lysosomal degradation in an autophagy dependent manner, but accumulate in large structures at early endosomes. Since our manuscript is already very dense and we presented already more data in the supplemental information as in main figures, we prefer not to include these data since they are redundant with data already present. Moreover, our new live cell experiments confirmed that a rapid and efficient fusion of TECPR1 structures with LC3C puncta occurs in NPCs, as explained in our response to the previous comment of this reviewer and documented in Supplemental movies 7, 8, 9 and 10.

Finally, this type of phenotype, which involves TECPR1-clustered autophagosomes attacking protein aggregates, must be examined by some type of CLEM. 

We entirely agree with the reviewer that visualizing how TECPR1 attacks protein aggregates. As already shown by CLEM in Figure S1e, TECPR1 localizes to lysosomes or autolysosomes. Moreover, we found that LC3C localizes to autophagosomes which contain electron dense material by CLEM (Figure 6a). To visualize both events independently with CLEM is already challenging, but a cross-correlation in these ultra-thin sections is very difficult. The major technical obstacle is that the fluorescence in ultra-thin sections is very weak compared to that in standard confocal fluorescence microscopy. Moreover, the likelihood to observe in the same cryo-section colocalizing fluorescence of GFP and RFP with organelles being cut such that they are nicely visible is very challenging. This is the reason way in most CLEM data, only one correlation of one fluorescence marker is used. We have, however, took on the challenge and provide here the requested information as seen in reviewer Figure 4. We even included in our analysis apart from TECPR1, the TECPR1∆PH-2xFYVE mutant.

**Reviewer Figure 6: Correlative light electron tomography of GFP-TECPR1 or GFP-TECPR1∆PH-2xFYVE and RFP-LC3C.**

Consistent with our single correlation data, RFP-LC3C is found on vesicles that contain electron dense material. These vesicles are in close contact with TECPR1 positive structures. In case of TECPR1 wildtype, these structures have autolysosome like morphology. To our surprise, structures that were TECPR1∆PH-2xFYVE positive did not show the typical morphology of early endosomes, but of multi-vesicular bodies. Together with our observation that these structures are EEA1-positive, we concluded that TECPR1∆PH-2xFYVE induces the formation of a mixed early-late endosomal population. Morover, as shown in the supporting figure 5 c and d in our manuscript, trafficking of endocytosed material to lysosomes is not impaired. This demonstrates that even though these endosomal structures possess an unusual morphology, they are fully functional.

Since this is a side aspect of our main massage and due to space limitations in the already very dense manuscript, we include these data as reviewer only figure.

Of minor concern:
-What happens to this system during starvation?

We initially performed all experiments in non-starved and starved cells. We did not observe significant differences which might be related to the fact that in HeLa cells high levels of non-selective autophagy are observed even in the absence of starvation. If we starve NSCs many cells detached from the substrate and were floating in the medium. The number of transfected cells under these conditions was extremely low and the cells were obviously suffering. We thus did not draw any conclusion from these data.

-It would be good to show some more experiments with endogenous LC3 upon TECPR1 overexpression at the beginning

We agree that experiments of endogenous LC3 with TECPR1 are useful. However, our manuscript contains already so many data and is very dense. Even the extensive analyses with many new interesting data for the revision of the manuscript could not be included in the manuscript.

-Is the TECPR1 construct missing the PH domain less punctate (Fig 4)?

No, we did not observe any significant differences between TECPR1 and TECPR1∆PH.

-Why is TECPR1 vacuolar in the RFP-2xFYVE overexpressing cells (Fig S4)?

Our CLEM experiments show that TECPR1 colocalizes to autolysosomes that are 500 nm in size (Fig S1e). Together with a strong colocalization of TECPR1 with LAMP2 and other lysosomal makers (such as HOPS complex subunit Vps41, data not shown), this suggests that TECPR1 localizes to lysosomes that already underwent fusion with autophagosomes. Moreover, we observed in NPCs that TECPR1 positive structures that already fused with LC3C puncta fuse with other LC3 puncta, suggesting that autolysosomes can receive multiple LC3C positive structures (Supplemental Movies 7 and 8). This explains why TECPR1 is frequently observed at bigger circular structures that have dimensions between 500 nm and 3 µm as shown in Fig S4.

-Does TECPR1 overexpression inhibit PI(4,5)P2 levels? In some of the photos it would appear to be so.

We did not observe strong difference in PI(4,5)P2 levels in cells that express TECPR1 and therefore did not study this aspect further. However, thanks to the comments of this reviewer, we did additional experiments in which we modulated the PI4P pool (see reviewer figure 4). Unexpectedly, we observed a strong colocalization of Sac2 with TECPR1 and LC3C, suggesting that PI4P is dephosphorylated at TECPR1/LC3C positive lysosomes. Knockdown of OCRL, which increases PI(4,5)P2 levels, had no effect on the colocalization of TECPR1 and LAMP2. Our new data suggest that PI4P is dephosphorylated at TECPR1/PI4P domains and that Sac2 thus modulates or restricts such domains, without impacting on the recruitment of LC3C to lysosomes.

**Reviewer comments, second version:**

Reviewers' comments:

Reviewer #1 (Remarks to the Author):

This is a revised manuscript. While the paper has been improved, I still have some concerns. Overall, I think that the model that has been proposed is supported by the data. On rereading, I am concerned that the authors have overstated the role of TECPR1-LC3C in aggregate clearance. P62 is likely to be associated with autophagosomes without being in aggregates and p62 binds many ubiquitinated proteins, which may also not be aggregates. In the absence of data formally showing that it is aggregates or even oligomers being cleared and that this process is specific to LC3C over other LC3s/ATG8s, I suggest that the authors rewrite this section to avoid committing to aggregates and possibly misleading readers.
There are also some technical issues and questions raised in the earlier round of reviews which have not been dealt with comprehensively:
1. Reviewer 1 asked for co-immunoprecipitation experiments. These have been provided for Fig 1d, but should also be shown for the data in 2D and ideally for comparisons of TECPR1 binding to LC3A, B and C. The reviewer gave LC3C-TECPR1 as an example, but there are clearly other interactions in the paper with Bio-ID that need biochemical confirmation.
2. The authors have not answered these questions posed by Reviewer 1 properly
a. Does TECPR1 reduce protein aggregates in cells where LC3C is knocked down? This means knockdown of TECPR1 or control knockdown in wild-type and LC3C knockdown/knockout cells. This is important as the data in the paper suggest that protein aggregate clearance should not be further impaired in LC3C knockdown cells when TECPR1 levels are reduced. It is important to show protein clearance data with knockdowns since most of the experiments shown are with TECPR1 overexpression, which may be misleading.
The authors’ new experiments to deal with the requests from reviewer 3 on p62/Ub clearance on blots were not successful and so they tried to use LC3C-TECPR1 interactions to support claims about protein clearance- however, these movies 7,,8,9,10 do not assess autophagy substrates – this makes the experiments suggested above even more important.
b. From the data in Reviewer Fig 2, I cannot see any difference in TECPR1 wt cells between control siRNA and siLC3C. These data need quantifying, controls blots showing levels of knockdown etc. and I think that the data from these two experiments should be in the paper itself.
c. Fig 6d – is this graph mislabelled? – it looks like the without Triton and with Triton colors are mixed up.


Reviewer #3 (Remarks to the Author):

The reviewer appreciate the authors’ efforts address the concerns. Under this stage, the reviewer believes that there may be a role of TECPR1 and LC3C in regulating auto-lysosomal fusion and aggrephagy. However the reviewer is still concerned with the strength of the data provided. Here are the suggestions to improve.
1. For all specific data to reviewers, please add them to the manuscript and discuss accordingly because they will significantly improve the quality of the work.
2. For the new data added:
1) The COIP in Fig.1b should be improved by comparing all isoforms of LC3 but not just LC3C. Because the BioID has shown some degree of TECPR1 with all six isoforms, validation of LC3C does not mean that it dominate over the other isoforms in the COIP assay. In addition, please beware of the band quality. As is shown in the current data, the LC3C-HA input band (on the right) is burned with a hole which is resulted from excessive antibody use or protein load.
2) For new protease protection data in Fig.6d, I do not see which band is quantified. Of the three visible bands vertically, I did not see the level of any of the band fits the quantification data.
3) For Reviewer Fig.2, please add the quantification because the reviewers are not convinced with just Snapshot (and guess the readers too).
4) For reviewer Fig.1. Wortmannin is misspelled in the figure. In addition, the review would suggest using the VPS34 specific inhibitors instead of PI3K pan inhibitor wortmannin because this may cause artifact derived from inhibition of other PI3Ks.
5) For reviewer Fig.3. Again please use VPS34 specific inhibitors to confirm. As in the Snapshot, it looks that the TECPR1 becomes smaller and more after wortmannin treatment. Could it be a non-specific effect caused by pan PI3K inhibition?
3. For the validation of the puncta formation of endogenous LC3C(review3 question No.2), reviewer understand that the problem of the current availability of LC3C specific antibodies. But the author should consider circumvent the problem by knocking in a Tag (e.g. a T7) to the LC3C gene and use the tag antibody instead. Or they could establish a stable cell line with comparable tagged LC3C expression to the endogenous LC3C to validate. Again this is essential to convince the reviewers and readers that what they have observed is not an artifact of LC3C overexpression.
4. For the pulse chase experiments suggested (review3 question No.3), the authors found difficult in performing in neuronal stem cells. But is the mechanism proposed specific for neuronal stem cells? If it is not, why not perform the experiments in other easily cultured cell line to validate?
5. For the MBL antibody (review3 question No.4), the reviewer is quite sure that this antibody is hard to recognize or at least reacts with LC3C much weaker than LC3B (at least 3-5 times less). This means that the majority of the puncta appear should be LC3B puncta. Therefore, it is hard to draw a conclusion of LC3C based on this antibody. The reviewer would suggest either remove the Figure and claim or repeat the experiment in cells with LC3A/B depletion. In this experiment, the author may consider another MBL antibody PM036, which has been confirmed to recognize LC3C both by the vendor and several other labs (even through still reduced binding compared to LC3A and B, but at least it could bind LC3C).
6. The reviewer is not convinced that the PMP70 antibody is working, even though the authors have done some improvement. I would suggest removing the figure.
7. For movie S2-4, please add the quantification into the manuscript and discuss accordingly.


Reviewer #4 (Remarks to the Author):

The authors have addressed the issues raised in my previous review in a comprehensive way. The one thing that puzzles me is that they did not incorporate any of the new experiments in either main or supplementary data. They treated the issues raised as a private matter between author and reviewer! I think they should consider adding some of the new data to the manuscript.

**Author rebuttal, second version:**

Reviewers' comments:

Reviewer #1 (Remarks to the Author):

This is a revised manuscript. While the paper has been improved, I still have some concerns. Overall, I think that the model that has been proposed is supported by the data. On rereading, I am concerned that the authors have overstated the role of TECPR1-LC3C in aggregate clearance. P62 is likely to be associated with autophagosomes without being in aggregates and p62 binds many ubiquitinated proteins, which may also not be aggregates. In the absence of data formally showing that it is aggregates or even oligomers being cleared and that this process is specific to LC3C over other LC3s/ATG8s, I suggest that the authors rewrite this section to avoid committing to aggregates and possibly misleading readers.

There are also some technical issues and questions raised in the earlier round of reviews which have not been dealt with comprehensively:

We thank the reviewer for reevaluating our revised manuscript. We agree that we did not formally demonstrated that the ubiquitinated p62 positive structures are protein aggregates in our previous manuscript. As suggested by this reviewer, it is essential to investigate whether LC3C is important and specifically required for the clearance of protein aggregates. In order to address this question, we have been working on two parallel approaches:

First, we have re-evaluated the function of LC3C in neural stem cells by performing a systematic and quantitative analysis of protein aggregation upon depletion of LC3C as well as a combined knock-down of all LC3 proteins or all ATG8 proteins. We treated these cells with puromycin to induce the formation of protein aggregates and quantified the number of Ub puncta. The data are shown in our new Figure 7c and d. We could demonstrate that the knock-down of LC3C alone leads to a significant increase in the number of Ub puncta per cell, implying that the clearance of protein aggregates is impaired. If we jointly knocked-down all LC3 proteins, no further accumulation of Ub puncta was observed. However a combined knock-down of all ATG8-protein members lead to a further increase in the number of Ub puncta. This suggests that LC3C and GABARAP proteins are acting independently, which is in agreement with previous studies that demonstrated that GABARAP proteins promote the fusion of autophagosomes and lysosomes. Our data indicate that LC3C promotes the recruitment of autophagosomes by TECPR1, which is upstream of the fusion event.

In light of the functional importance of TECPR1 in NPCs (Figure 7a and S7) and the selective interaction of TECPR1 with LC3C in vitro and in vivo, all data in our manuscript strongly support the existence of a specific TECPR1/LC3C mediated autophagic pathway that facilitates the clearance of protein aggregates at least under conditions of augmented aggregation.

Second, to proof that the clearance of protein aggregates is enhanced in TECPR1 expressing neurons, we performed new experiments in which we overexpressed the disease related protein Huntingtin Q97 (expansion of the glutamine repeat). The expression plasmid was a gift from F. Ulrich Hartl (MPI of Biochemistry, Martinsried, Germany) who also helped us to set up experiments and to interpret our data. We found that in cells that did not expressed TECPR1, HttQ97 was forming aggregates that colocalized with ubiquitin. However, cells that expressed TECPR1 were devoid of such aggregates. Furthermore, HttQ97 aggregates accumulated in NSCs upon expression of TECPR1∆PH2xFYVE in bright puncta that colocalized with ubiquitin and TECPR1∆PH2xFYVE. Upon inhibition of lysosomal degradation by BafA1 treatment, HttQ97 aggregates were present in all cells, independently of the expressed TECPR1 variant.

Collectively, we think our new experiments are now providing enough evidence that supported our claim that TECPR1 promotes clearance of protein aggregates in an autophagy dependent manner.

Reviewer 1 asked for co-immunoprecipitation experiments. These have been provided for Fig 1d, but should also be shown for the data in 2D and ideally for comparisons of TECPR1 binding to LC3A, B and C. The reviewer gave LC3C-TECPR1 as an example, but there are clearly other interactions in the paper with Bio-ID that need biochemical confirmation.

We entirely agree with the reviewer that biochemical confirmation of the specific interaction of LC3C with TECPR1 in vivo would strengthen our conclusion. We therefore compared interactions of TECPR1 with all ATG8 proteins by co-immunoprecipitation as shown in our new Figure 1b. Consistent with our experiments in vitro and our proximity biotinylation assay, our experiments confirm the specificity of TECPR1 for LC3C compared to other ATG8 family members. We also performed by co-immunoprecipitation experiments of the TECPR1-LIR mutant with LC3B and LC3C to confirm our previous BioID experiments (new Figure S2c).

2. The authors have not answered these questions posed by Reviewer 1 properly

a. Does TECPR1 reduce protein aggregates in cells where LC3C is knocked down? This means knockdown of TECPR1 or control knockdown in wild-type and LC3C knockdown/knockout cells. This is important as the data in the paper suggest that protein aggregate clearance should not be further impaired in LC3C knockdown cells when TECPR1 levels are reduced. It is important to show protein clearance data with knockdowns since most of the experiments shown are with TECPR1 overexpression, which may be misleading.

We agree with the reviewer and repeated these pulse chase experiments in a systematic fashion. We provided necessary quantifications as requested also by reviewer 2 and integrated all new data in the manuscript (Figure S6c, d, e, f). We found that the clearance of protein aggregates is not significantly affected in cells in which TECPR1 has been knocked out. This is consistent with previous reports that demonstrated that other factors including the HOPS complex tether autophagosomes to lysosomes to mediate fusion. Such interactions can thus bypass TECPR1 mediated recruitment. If we, however, deplete LC3C in TECPR1 knockout cells, we observed a mild defect in the clearance of protein aggregates (Figure S6d and new Figure S6e). The strongest effect is, however, observed if GABARAPs are silenced, which is consistent with previous reports that GABARAPs promote fusion of autophagosomes and lysosomes and can partially compensate depletion of LC3 proteins.

To our surprise, this picture changed when we compared the number of Ub puncta in puromycin treated NPCs in which LC3C was knocked-down. In these cells, a significant increase in the number of Ub puncta was apparent, which was not further increased by combined knock-down of all three LC3 proteins. Only in combination with a knock-down of GABARAP proteins, a further increase in the number of Ub puncta was observed. This is consistent with previous reports that GABARAPs are involved in the fusion of autophagosomes and lysosomes. Together with our observation that overexpression of TECPR1 leads to a significant decrease in the number of Ub puncta in puromycin treated NSCs, but not in HeLa cells, our data now strongly support the notion that TECPR1 and LC3C are facilitating the clearance of protein aggregates in neural stem cells, whereas other, complementary mechanisms are masking this effect in HeLa cells. Similar cell-type specific autophagic activities have been reported previously, underscoring the importance to investigate protein functions in context of different cell types. Moreover, we investigated aggregation and clearance of the disease related protein HttQ97 as already mentioned. In these experiments we observed that HttQ97 aggregates were present in NSCs and in TECPR1∆PH2xFYVE expressing cells, but not in cells that expressed TECPR1. The augmented clearance of these aggregates depended on autophagy since aggregates were present upon inhibition of lysosomal degradation by BafA1.

The authors’ new experiments to deal with the requests from reviewer 3 on p62/Ub clearance on blots were not successful and so they tried to use LC3C-TECPR1 interactions to support claims about protein clearance- however, these movies 7,,8,9,10 do not assess autophagy substrates – this makes the experiments suggested above even more important.

We agree with the reviewer and performed now protein clearance experiments in NPCs. The data of this new study are included in the manuscript (new Figure 7c, d and e).

b. From the data in Reviewer Fig 2, I cannot see any difference in TECPR1 wt cells between control siRNA and siLC3C. These data need quantifying, controls blots showing levels of knockdown etc. and I think that the data from these two experiments should be in the paper itself.

We thank the reviewer for suggesting that the data should be integrated into the manuscript. We therefore included them as new Figures S6c, d, e, f, g and h. We also quantified data as suggested by the reviewer as shown in Figure S6e and h. We agree with the reviewer that there is no obvious difference in the number of aggregates in LC3C knock down cells. The quantification revealed that only a combined knock out of TECPR1 with siRNA of LC3C lead to a slightly impaired clearance of protein aggregates (Figure S6e). As already discussed, a remarkable different phenotype was observed in neural stem cells in which a statistically significant increase in Ub puncta was observed upon knock-down of LC3C whereas a combined knock-down of all LC3 proteins did not further increase the number of Ub-puncta. This is consistent with the observation that overexpression of TECPR1 strongly diminishes the formation of protein aggregates in NSCs but not in HeLa cells. Taken together, our data strongly suggest that TECPR1 and LC3C cooperate to promote clearance of protein aggregates and that this pathway is important in neural stem cells, whereas in HeLa cells compensatory mechanisms exist.

c. Fig 6d – is this graph mislabelled? – it looks like the without Triton and with Triton colors are mixed up.

Yes, indeed. We thank the reviewer for alerting us that the samples were mislabeled and corrected our mistake in the new Figure 6d.

Reviewer #3 (Remarks to the Author):

The reviewer appreciate the authors’ efforts address the concerns. Under this stage, the reviewer believes that there may be a role of TECPR1 and LC3C in regulating auto-lysosomal fusion and aggrephagy. However the reviewer is still concerned with the strength of the data provided. Here are the suggestions to improve.

We thank the reviewer for appreciating our effort. Motivated by the insightful comments of this reviewer, we made addition efforts to improve our manuscript further and to address the remaining concerns as outlined below.

1. For all specific data to reviewers, please add them to the manuscript and discuss accordingly because they will significantly improve the quality of the work.

We thank the reviewer for appreciating the data of our first revision. We have now included these data into the manuscript and discussed them as suggested.

2. For the new data added:

1) The COIP in Fig.1b should be improved by comparing all isoforms of LC3 but not just LC3C. Because the BioID has shown some degree of TECPR1 with all six isoforms, validation of LC3C does not mean that it dominate over the other isoforms in the COIP assay. In addition, please beware of the band quality. As is shown in the current data, the LC3C-HA input band (on the right) is burned with a hole which is resulted from excessive antibody use or protein load.

We thank the reviewer for this comment. We have repeated co-IP experiments with all ATG8 isoforms as requested. Furthermore, we performed additional co-IPs in which we compared binding of the TECPR1 LIR mutant to LC3C and LC3B to confirm our corresponding BioID experiments. We included these new data in our manuscript as new Figure 1b and new Figure S2c.

2) For new protease protection data in Fig.6d, I do not see which band is quantified. Of the three visible bands vertically, I did not see the level of any of the band fits the quantification data.

We thank the reviewer for the comment and entirely agree that the bands that we quantified in the blots were not obvious. We have improved the Figure by showing the band that has been quantified specifically in Figure 6d and the full blot with indicated areas that have been cut out for Figure 6d in the Source Data File.

3) For Reviewer Fig.2, please add the quantification because the reviewers are not convinced with just Snapshot (and guess the readers too).

We thank the reviewer for this comment. Since all reviewers invited us to include the reviewer only data into the manuscript, we have performed additional experiments and did the requested quantifications which are now shown in Figure S6c, d, e, f, g and f. Notably, we expanded the study in which we analyzed the effect of ATG8 depletion in combination with TECPR1 deletion on the number of Ub puncta. We now include data for KD of GABARAPs in Figure S6e. Together with our new experiments on protein aggregate clearance in neural stem cells, we obtained much better insights into the functional relevance of TECPR1 and LC3C. Notably, we observed that neural cells are much more sensitive to LC3C depletion compared to HeLa cells. This is in line with our observation that overexpression of TECPR1 in HeLa cells has no big impact on the number of protein aggregates, whereas TECPR1 expression in neural stem cells let to a strong decline of protein aggregates.

4) For reviewer Fig.1. Wortmannin is misspelled in the figure. In addition, the review would suggest using the VPS34 specific inhibitors instead of PI3K pan inhibitor wortmannin because this may cause artifact derived from inhibition of other PI3Ks.

We thank the reviewer for the notification that we misspelled Wortmannin. In addition, we repeated these experiments using the VPS34 specific inhibitor SAR405 as requested by the reviewer. Notably, we observed that the localization of TECPR1 with LAMP1 and Lysotracker is strongly affected in SAR405 treated cells. This suggests that inhibition of autophagy has an effect on the localization of TECPR1. Furthermore, we found that at an ultrastructural level, TECPR1 co-localizes with autolysosomes instead of lysosomes (Figure S1e and new Figure S1g) and TECPR1 structures are fusing with several LC3C puncta in life cell microscopy experiments. Combined, these data suggest that the dependency of lysosomal localization of TECPR1 on autophagy is related to its recruitment to autolysosomes.

5) For reviewer Fig.3. Again please use VPS34 specific inhibitors to confirm. As in the Snapshot, it looks that the TECPR1 becomes smaller and more after wortmannin treatment. Could it be a non-specific effect caused by pan PI3K inhibition?

We thank this reviewer for paying close attention to our data and for suggesting to use a more selective inhibitor. From our new experiments in which we used SAR405 instead of Wortmannin, we observed that the localization of TECPR1 is indeed affected if autophagy is inhibited. We show these new data in our new Figure S6i and discussed this observation in the updated manuscript text.

3. For the validation of the puncta formation of endogenous LC3C(review3 question No.2), reviewer understand that the problem of the current availability of LC3C specific antibodies. But the author should consider circumvent the problem by knocking in a Tag (e.g. a T7) to the LC3C gene and use the tag antibody instead. Or they could establish a stable cell line with comparable tagged LC3C expression to the endogenous LC3C to validate. Again this is essential to convince the reviewers and readers that what they have observed is not an artifact of LC3C overexpression.

We entirely agree with the Reviewer that overexpression is always affecting the experimental system and precaution needs to be taken when such data are interpreted. We thank the reviewer for the excellent suggestion to use for example CRISPR Cas9 technology to establish cell lines that express tagged ATG8s at native protein levels. We are in the process of setting these experiments up. However, this is a very elaborated and time consuming procedure. To validate our observation in the present study, we performed additional experiments in which we knocked LC3C down to observe the effect on the clearance of protein aggregates. Our data confirm that LC3C is important for aggrephagy in NSCs, but dispensable in HeLa cells. This is a surprising result but in agreement with several recent studies in which a similar cell type dependent difference of experimental results have been observed. To validate our data regarding the recruitment of LC3C by TECPR1, we again not only rely on colocalization experiments in cells overexpressing these proteins. We combined our analysis with in vitro reconstitutions in which we used purified proteins in well defined systems to show that TECPR1 indeed directly and physically interact with LC3C and that this interaction is selective. Finally, we combined in vivo colocalizations of TECPR1 with our endosomal targeted TECPR1∆PH 2xFYVE mutant. These experiments were important because they demonstrated that even under condition of overexpressed LC3C, the majority of LC3C puncta are not reaching lysosomal compartments but are re-directed to early endosomes. Again, the combination of different approaches allowed us to exclude that the observed effects are a sole cause of overexpressing LC3C.

4. For the pulse chase experiments suggested (review3 question No.3), the authors found difficult in performing in neuronal stem cells. But is the mechanism proposed specific for neuronal stem cells? If it is not, why not perform the experiments in other easily cultured cell line to validate?

We thank the reviewer for this suggestion. We do think that the pathway is cell type dependent and are now providing compelling evidence to substantialize this claim. As already eluted to, we did not observe strong effects on the clearance of proteins aggregates in HeLa cells. However, our new data in NSCs show that LC3C depletion impairs the degradation of protein aggregates. Furthermore, overexpression of TECPR1 did not facilitate clearance of protein aggregates in HeLa cells whereas a strongly facilitated autophagy-dependent clearance was induced in NSCs. We examined various neural cell lines in terms of transfection efficiency as this was the major bottleneck in setting up quantitative biochemical assays. However, transfection efficiencies were always well below 50%, which imposes the same difficulty as encountered in NSCs.

5. For the MBL antibody (review3 question No.4), the reviewer is quite sure that this antibody is hard to recognize or at least reacts with LC3C much weaker than LC3B (at least 3-5 times less). This means that the majority of the puncta appear should be LC3B puncta. Therefore, it is hard to draw a conclusion of LC3C based on this antibody. The reviewer would suggest either remove the Figure and claim or repeat the experiment in cells with LC3A/B depletion. In this experiment, the author may consider another MBL antibody PM036, which has been confirmed to recognize LC3C both by the vendor and several other labs (even through still reduced binding compared to LC3A and B, but at least it could bind LC3C).

We entirely agree with the reviewer that only based on this one experiment, a conclusion regarding LC3C selectivity cannot be drawn. The major concern of this reviewer was the claim that LC3C is selectively recognized by TECPR1 and that the LIR motif contributes to recognition but is not essential. In the corresponding Figure (now Figure S5f), we observed a colocalization of LC3 puncta with TECPR1 wt but not with the corresponding LIR mutants. In Figure S5e we showed that only LC3C strongly colocalized with TECPR1∆PH2xFYVE, demonstrating that even in condition in which LC3B is overexpressed, its colocalization with TECPR1 is weak. This observation was also confirmed by our co-IP experiments of TECPR1 with all ATG8 variants (new Figure 1b) which shows that binding of LC3C to TECPR1 is much stronger than that of the other ATG8s and by our BioID experiments, which showed similar results. Altogether, we are convinced that using the LC3 antibody in this Figure is not misleading as the point we wanted to make was that there is a strongly reduced colocalization of TECPR1 LIR mutants with autophagosomes. In agreement with this we see many LC3 puncta. Of course, some of them correspond to LC3B puncta but our quantitative analysis regarding the abundance of ATG8 puncta in non-starved cells revealed that LC3C puncta are much more abundant, independently of the transported cargo or the nature of ATG8 puncta (LC3B ≈ 10 puncta per cell, LC3B ≈ 60 puncta per cell).

6. The reviewer is not convinced that the PMP70 antibody is working, even though the authors have done some improvement. I would suggest removing the figure.

We agree with the reviewer that the staining using the PMP70 antibody is not strong. We, however, compared our staining with that of references (Sigma Aldrich, Merck, Abcam) and found identical staining quality. We therefore combined immunofluorescence with correlative light electron microscopy to identify cargo that is enclosed by LC3C positive autophagosomes. We could demonstrate that LC3C autophagosomes are not containing defined cytoplasmic structures such as organelles. We observed instead that they contain electron dense amorphous material (Figure 6a and new Figure S1g). We therefore suggest to retain the Figure because we do see peroxisomal staining that is not colocalizing with LC3C in immunofluorescence and consolidated our findings with complementary ultrastructural data.

7. For movie S2-4, please add the quantification into the manuscript and discuss accordingly.

We included the quantification into the manuscript and updated the section accordingly.

Reviewer #4 (Remarks to the Author):

The authors have addressed the issues raised in my previous review in a comprehensive way. The one thing that puzzles me is that they did not incorporate any of the new experiments in either main or supplementary data. They treated the issues raised as a private matter between author and reviewer! I think they should consider adding some of the new data to the manuscript.

We thank the reviewer for acknowledging our efforts to address all the concerns raised. We indeed performed many new experiments for the first round of revision and even additional a number of further experiments for the second round. The reason why we were not including all the data into the manuscript was that the original manuscript was already very dense. We, however, thank the reviewer for motivating us to include such data into the manuscript. Due to space limitations, most of them are now presented as Supplementary Figures.

**Reviewer comments, third version:**

REVIEWERS' COMMENTS:

Reviewer #1 (Remarks to the Author):

I am satisfied with the revisions


Reviewer #3 (Remarks to the Author):

The authors have addressed my concerns.

**Author rebuttal, third version:**
